# Rational Design of Zwitterionic Polymers with Tunable Phase Separation Propensity

**DOI:** 10.1021/acs.macromol.5c01394

**Published:** 2025-10-08

**Authors:** Timo N. Schneider, Suiying Ye, Nicola Carrara, Umberto Capasso Palmiero, Matteo Salvalaglio, Paolo Arosio

**Affiliations:** † Department of Chemistry and Applied Biosciences, 27219 ETH Zurich, Vladimir Prelog Weg 1, Zurich 8093, Switzerland; ‡ Department of Chemical Engineering, 4919 University College London, London WC1E 6BT, U.K.

## Abstract

Zwitterionic polymers are emerging as promising candidates for forming fluid-like coacervates with desirable characteristics, including antifouling capabilities, stimulus responsiveness, and biocompatibility. These attributes make them particularly appealing for applications in the biomedical field, including bioseparation, biochemical analysis, and diagnostics. However, there are currently no clear guiding principles for predicting the phase separation behavior of zwitterionic polymers and informing the design of novel phase-separating polymers. In this study, we develop a workflow that combines molecular dynamics simulations, theory, and experiments to predict the phase separation propensity of zwitterionic polymers, as well as the material properties of the resulting coacervates. We validate our simulation-based workflow as a predictive tool by synthesizing new zwitterionic polymers that undergo no phase separation, liquid–liquid phase separation, or liquid–gel phase separation. Beyond their predictive power, we show that molecular simulations provide insights into the attractive homotypic intermolecular interactions mediated by distinct functional groups, rationalizing the large differences observed between zwitterionic monomers that exhibit minimal structural variations. Our approach provides valuable insights into the molecular principles governing the phase separation of distinct zwitterionic polymers, with important implications for the design of their materials.

## Graphical abstract

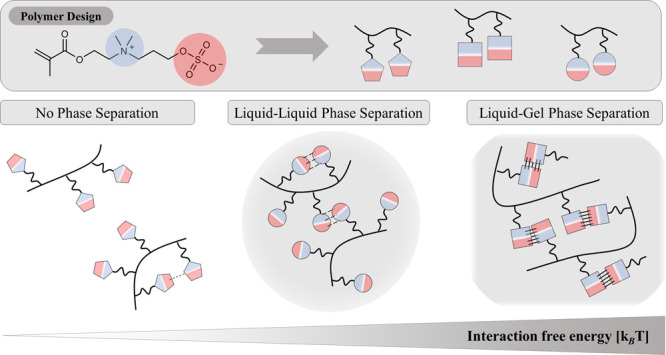

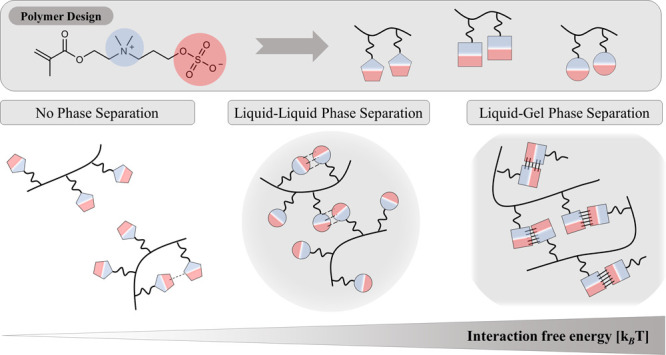

## Introduction

Polymeric coacervates represent an important class of materials with diverse applications in the biomedical sector, as well as in the chemical and food industries, and as mimics of biological systems.^1–10^ Examples include complex coacervates formed from two oppositely charged polymers, as well as simple coacervates based on poly­(*N*-isopropylacrylamide) (PNIPAM), where coacervation is driven by temperature-dependent hydrophobic interactions, resulting in lower critical solution temperature (LCST) behavior.^11–13^

Recently, it has been demonstrated that zwitterionic polymers based on sulfabetaine methacrylate can also form liquid-like coacervates.^14,15^ Zwitterionic polymers carry positive and negative charges, which mediate attractive electrostatic interactions between different chains. In contrast to PNIPAM, the phase separation of zwitterionic polymers is enthalpically driven and exhibits an upper critical solution temperature (UCST). These materials are expected to be gentler toward biomolecules such as proteins since the absence of hydrophobic interactions minimizes the risk of unwanted structural changes.^15–17^ Moreover, the dependence of coacervation on electrostatic interactions confers responsiveness also to ionic strength and pH, in addition to temperature.^3,18,19^ Importantly, zwitterionic polymers have demonstrated exceptionally low nonspecific molecular adsorption, which, together with their biocompatibility, promotes their use in various biomedical fields, including drug delivery, biosensors, and bioseparation.^20–26^

However, not all zwitterionic polymers undergo phase separation. For instance, the polymers formed with sulfobetaine methacrylate are significantly more soluble in water than chains based on sulfabetaine methacrylate, despite the minimal structural differences between the two monomers.^15^ For the design of smart materials, it is crucial to understand the intermolecular interactions at the molecular level. This will also lead to the identification of novel zwitterionic polymers capable of undergoing liquid–liquid phase separation and the design of zwitterionic coacervates with tunable stimuli responsiveness and adjustable material properties.

These operations are challenging due to the lack of a theoretical framework. While simple guiding rules such as differences in anionic and cationic charge densities are able to rationalize the varying self-association of sulfobetaine and carboxybetaine polymers,^27,28^ no clear rules have emerged in other cases. For instance, increasing the carbon spacer length in different parts of sulfobetaine can have opposing effects on phase separation propensity.^29^ On the other hand, molecular dynamics (MD) simulations are a powerful tool for assessing the behavior of new molecules and providing insights into nanoscale interactions. In the context of zwitterionic polymers, MD has helped study hydration states, conformational properties, ion association, protein interactions, and self-association.^30–34^

Simulating a phase-separating polymer system at an atomistic resolution poses computational challenges due to the system size and time scales involved, especially when simulating many different polymers. In this study, we applied MD simulations to quantify the strength of homotypic intermolecular interactions between individual monomers using this as a computationally accessible predictor of the phase separation behavior of the corresponding polymers. This simplification inevitably overlooks other potentially important factors such as backbone connectivity, many-body interactions, or chain-end effects. Still, several analytical models based on monomer interactions are reasonably consistent with experimental data on polymer phase separation.^35–37^

We validated our computational predictions with experiments, demonstrating that our strategy yields zwitterionic polymers with tunable phase separation and stimulus responsiveness. Moreover, our MD simulations unraveled molecular details of the homotypic intermolecular interactions responsible for polymer coacervation, therefore rationalizing the large differences observed between polymers composed of structurally similar monomers.

## Results and Discussion

### Design of Novel Zwitterionic Polymers with Different Phase Separation Propensities

Previous studies have demonstrated that the phase separation of zwitterionic polymers can be driven by electrostatic interactions mediated by sulfabetaine methacrylate groups, which can be conceptualized as “stickers.” ^14,15,38–40^ Starting from this reference sticker, we designed a set of monomers characterized by structural variations from the sulfabetaine methacrylate (ZB) monomer.^15^ We followed strategies that have been shown to affect the thermoresponsive behaviors of poly­(sulfobetaine methacrylates).^29,41^ For instance, we changed the alkyl chain length between the ammonium and the sulfate group (ZB2), the alkyl chain length of the substituent group on the ammonium nitrogen (ZB3), or the substituent group on the ammonium nitrogen (changing a methyl to a phenyl group in the case of ZB5) (Figure 1A). We also considered the sulfobetaine methacrylate (SB), which exhibits lower homotypic interactions and has been shown to decrease the cloud point temperature upon copolymerization with ZB monomers.^3,14,42^ Furthermore, the zwitterionic monomers carboxybetaine methacrylate (CB) and 2-methacryloyloxyethyl-phosphorylcholine (MPC) were also analyzed. The complete list of the 27 distinct monomers considered in this work is shown in Table S1.

**Figure 1:**
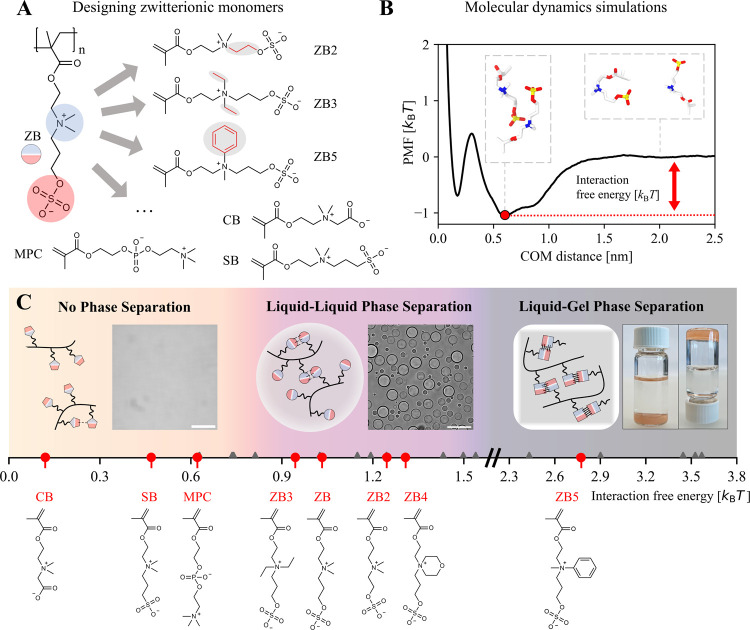
Design of zwitterionic polymers with different phase separation propensities. (A) Starting from the reference ZB monomer, we designed monomers with distinct structural variations, and monomer–monomer interactions were analyzed in silico. (B) Potential of mean force (PMF) profile obtained from Umbrella sampling for homotypic ZB–ZB interactions. The depth of the free energy minimum was considered to be a measure of the interaction free energy. Following this approach, homotypic interactions were quantified for the entire set of 27 monomers. (C) Overview of different phase separation behaviors as a function of interaction strength between monomers. Experimentally validated predictions are marked in red with the corresponding chemical structures below. Insets: microscopic image of polySB at 0.25 mg/mL in 150 mM NaCl (left); microscopic image of polyZB3 at 0.25 mg/mL in 150 mM NaCl (middle); photograph of polyZB5 in 3 M NaCl forming an undissolved gel (right). Scale bar: 50 μm.

We quantified the strength of the homotypic intermolecular interactions between these 27 distinct monomers by MD simulations. We used the GAFF2 ^43^ force field with the extended simple point charge (SPC/E) water model^44^ due to its ability to capture the dipole moment and dielectric constant of water molecules. The atomistic partial charges were calculated using the restrained electrostatic potential (RESP) method, based on electrostatic potentials obtained from Hartree–Fock (HF/6-31G*) calculations in vacuo. Using Umbrella sampling (US),^45,46^ we evaluated the potential of mean force (PMF) as a function of the center-of-mass (COM) distance between two monomers. The depth of the minimum within the main well of the PMF profile was selected as a measure of the interaction free energy (Figure 1B). Simulations were carried out without any orientational restraints on the molecules, enabling a thorough exploration of the different interaction modes. Additional details on the computational procedure are described in the methods section. For the different monomers, we calculated different free energies of interaction in the range from 0.12 to 4.60 *k*_B_ *T* at 300 K (Table S1). Specifically, the interaction free energies of most ZB variants were around 1 *k*_B_ *T*, while the values for CB, SB, and MPC were significantly lower (0.12–0.62 *k*_B_ *T*).

To validate predictions based on molecular simulations, we synthesized homopolymers with a degree of polymerization of 200 by reversible addition–fragmentation chain transfer (RAFT) polymerization for a subset of 8 monomers. The phase separation behavior of these polymers was characterized by bright-field microscopy at room temperature. Most polymers based on ZB monomer variants (polyZB, polyZB2, polyZB3, and polyZB4) formed coacervates in 150 mM NaCl aqueous solution, corresponding to physiological levels,^47^ at a polymer concentration of 0.25 mg/mL. In contrast, we observed no coacervation of polyCB and polySB and no significant coacervation of polyMPC (Figures 1C and S2). It is important to note that the exact boundaries between the regimes also depend on the total polymer as well as salt concentration. For instance, polySB also forms small coacervates at very high concentrations (Figure S3). On the other hand, phase separation behavior in 0 mM NaCl aqueous solution was comparable to that at 150 mM (Figure S4). The molecular simulations predicted strong homotypic interactions between the ZB5 monomers (2.77 *k*_B_ *T*). Indeed, the polymerization of this monomer led to the formation of a gel in the reaction mixture, which remained undissolved, even after several days of incubation in a 3 M NaCl aqueous solution (Figure 1C). The gel remained stable for more than 10 months of incubation (Figure S5).

We note that liquid–liquid phase separation can be coupled to percolation.^48^ In our framework, the transition from liquid–liquid to liquid–gel phase separation represents a continuous shift from a weak to a strong physical gel governed by the increasing interaction strength of the polymer chains in the dense phase.^35,48^ With the corresponding increase of interaction lifetime, gel-like behavior becomes dominant also at longer time scales, as observed for the ZB5 polymer.

In summary, the experimental data demonstrated that MD can guide the design of new zwitterionic homopolymers that undergo distinct phase separation behaviors. To further validate the approach more quantitatively, we tested the model’s ability to describe the dependence of phase separation of different homopolymers on salt concentration. Phase separation of zwitterionic polymers is inhibited at high salt concentrations due to the suppression of attractive electrostatic interactions caused by charge screening^49^ (Figure 2A). We prepared solutions of various homopolymers at a constant polymer concentration of *C*_p0_ = 0.25 mg/mL with increasing salt concentrations. We then measured the maximum salt concentration (*C*_s,max_) at which phase separation could still be detected using a standard bright-field microscope. We observed different values of *C*_s,max_ for different homopolymers (Figure 2B), with polyZB2 exhibiting the highest resistance of phase separation toward salt addition. Despite this strong phase separation propensity, its coacervates behaved liquid-like as indicated by the short coalescence time scale (Figure 2C). Even though the structural differences in the corresponding monomers are minimal, the different phase separation propensities of polymers correlated well with the free energy of interaction quantified in silico (Figure 2B). These results demonstrate that our strategy can identify new zwitterionic polymers undergoing either liquid–liquid phase separation or a liquid–gel transition.

**Figure 2:**
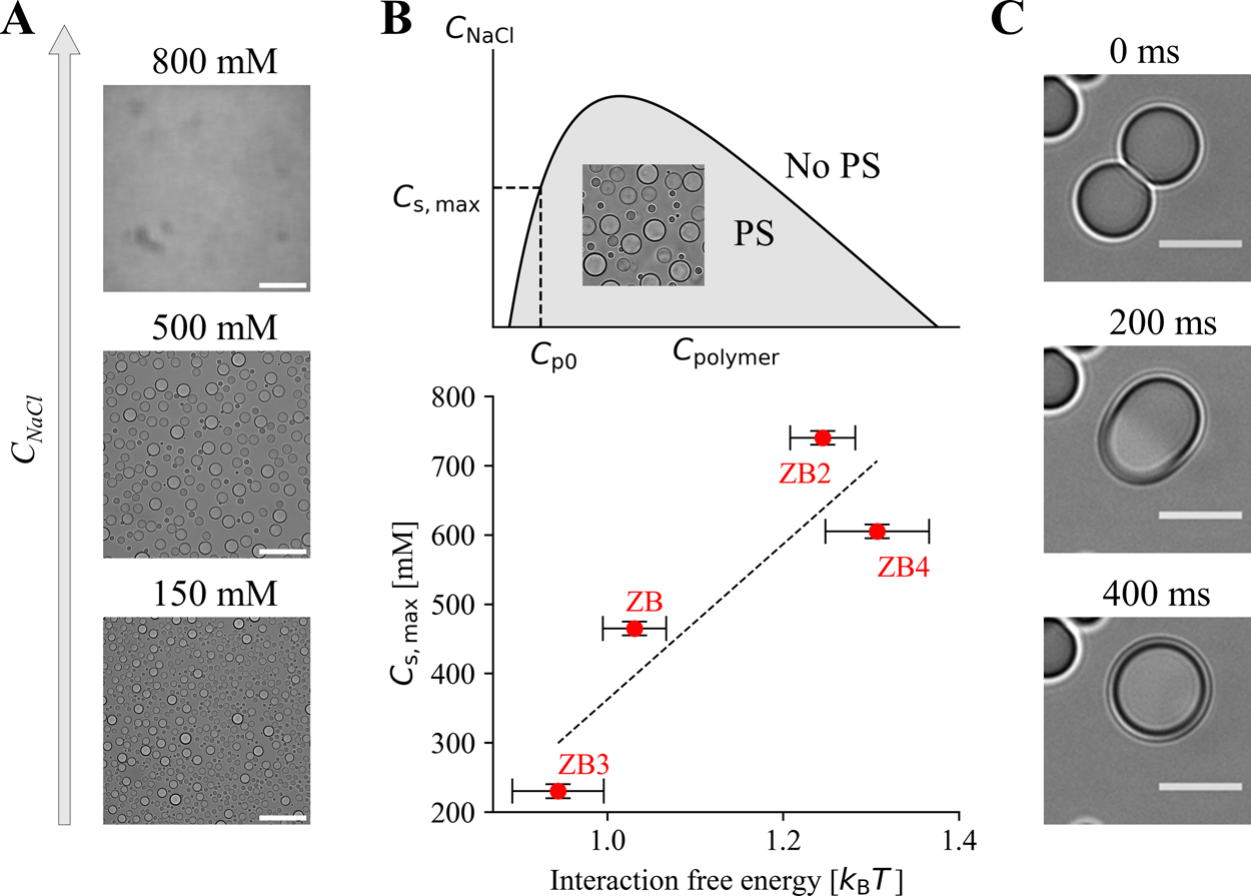
(A) Decreasing volume fraction of the coacervate phase of polyZB4 with increasing salt concentration. Scale bar: 50 μm. (B) Maximum sodium chloride concentration at which phase separation was still observed (*C*_s,max_) for different homopolymers at a fixed polymer concentration *C*_p0_ of 0.25 mg/mL. The measured maximum salt concentration correlates with the free energy of interaction predicted by MD. (C) Fast droplet fusion illustrates the liquid-like character of polyZB2 coacervates. Scale bar: 5 μm.

### Enhanced Tunability by Copolymerization

The gel phase formed by polyZB5 could not be dissolved at the highest tested salt concentration (5 M) and was therefore not included in Figure 2. To compare the phase separation propensity of polymers based on ZB5 with that of the other zwitterionic polymers, we generated copolymers containing various ratios of ZB5 and SB at a constant total degree of polymerization (Table S3). Both homotypic SB–SB interactions and heterotypic interactions between SB and ZB variants are weaker than homotypic ZB5–ZB5 interactions (Tables S1 and S2), and therefore, incorporating SB reduces phase separation propensity. ZB–SB copolymers can be described by the “stickers-and-spacers” framework, in which ZB groups mediate the attractive intermolecular interactions driving phase separation, while SB monomers behave as spacers intercalated among the stickers, modifying solubility and other properties of the coacervates.^38–40^ Upon dilution with the SB monomer, ZB5-SB copolymers indeed formed liquid-like, micrometer-sized coacervates, as observed under bright-field microscopy (Figure S6). These coacervates displayed reversible dissolution and reformation during heating and cooling cycles (Figure S7).

We characterized the phase separation of different copolymers in terms of the maximum salt concentration where phase separation was still observed (*C*_s,max_), and cloud point temperature *T*_cp_ at a fixed polymer concentration of 0.25 mg/mL and salt concentration of 150 mM. As expected, *C*_s,max_ and *T*_cp_ decreased upon increasing the fraction of the SB monomer. For all synthesized polymers, a linear relationship was observed between the square root of *C*_s,max_ and the fraction of sticker monomers in the polymers (*f*_sticker_) (Figure 3A). This linear relationship was also observed in other copolymers formed with ZB and either CB or MPC as spacers (Figure S8). The values of *C*_s,max_ for the various copolymers generally increased with the strength of sticker–sticker interactions, consistent with the predictions based on molecular simulations (Figure 3A and Table S1).

**Figure 3:**
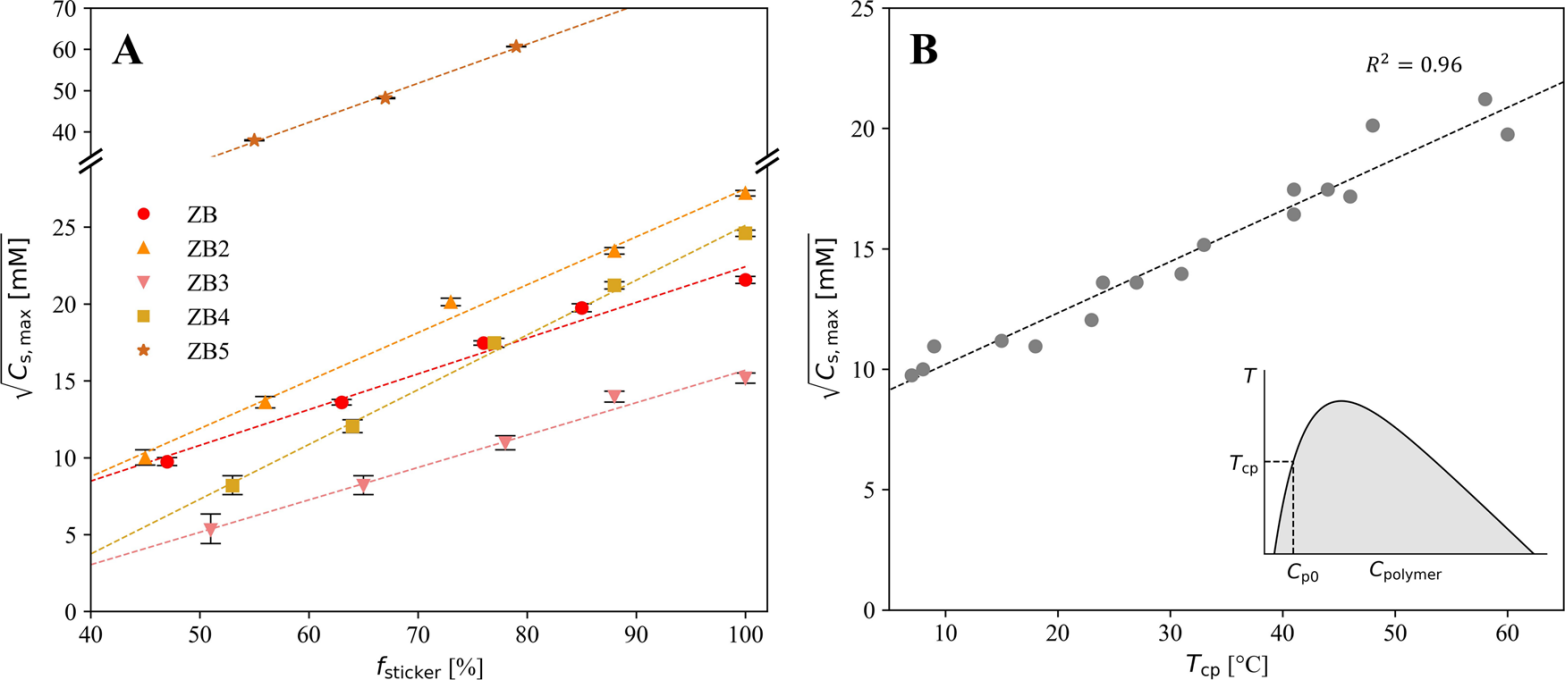
(A) Measured $\sqrt{C_{s,\max}}$ for different copolymers containing increasing fractions of SB monomer. (B) Linear relationship between the cloud point temperatures and $\sqrt{C_{s,\max}}$ measured for all polymers.

Interestingly, the measured cloud point temperatures *T*_cp_ of the different homo- and copolymers also correlated linearly with the measured $\sqrt{C_{s,\max}}$ for all tested zwitterionic polymers (Figure 3B). Overall, these linear relationships can facilitate the rational design of copolymers with a tailored phase separation behavior.

### Unraveling Molecular Interactions Underlying Phase Separation

The results shown in the previous paragraphs have demonstrated that our procedure can guide the design of zwitterionic homopolymers that undergo distinct phase separation behaviors. Moreover, the in silico analysis can offer molecular insights into homotypic interactions, rationalizing the differences observed among different monomers. We analyzed the configurations of homotypic ZB, SB, and ZB5 interactions using the Umbrella sampling trajectories. These monomers were selected as representative examples spanning a broad range of interaction strengths (Figure 4A). To reduce the dimensionality of the conformational ensembles, we performed a principal component analysis (PCA) with a set of interatomic distances as input variables using a total of 30,000 frames per monomer. Assigning appropriate weights to the frames ultimately resulted in a probability distribution or, equivalently, a free energy surface in a two-dimensional space. We identified local minima on the free energy surface and assigned corresponding free energy values, quantifying the relative stability of the conformations (see Methods for details). Sampling frames around these minima revealed distinct modes of interaction (Figure 4B–D).

**Figure 4:**
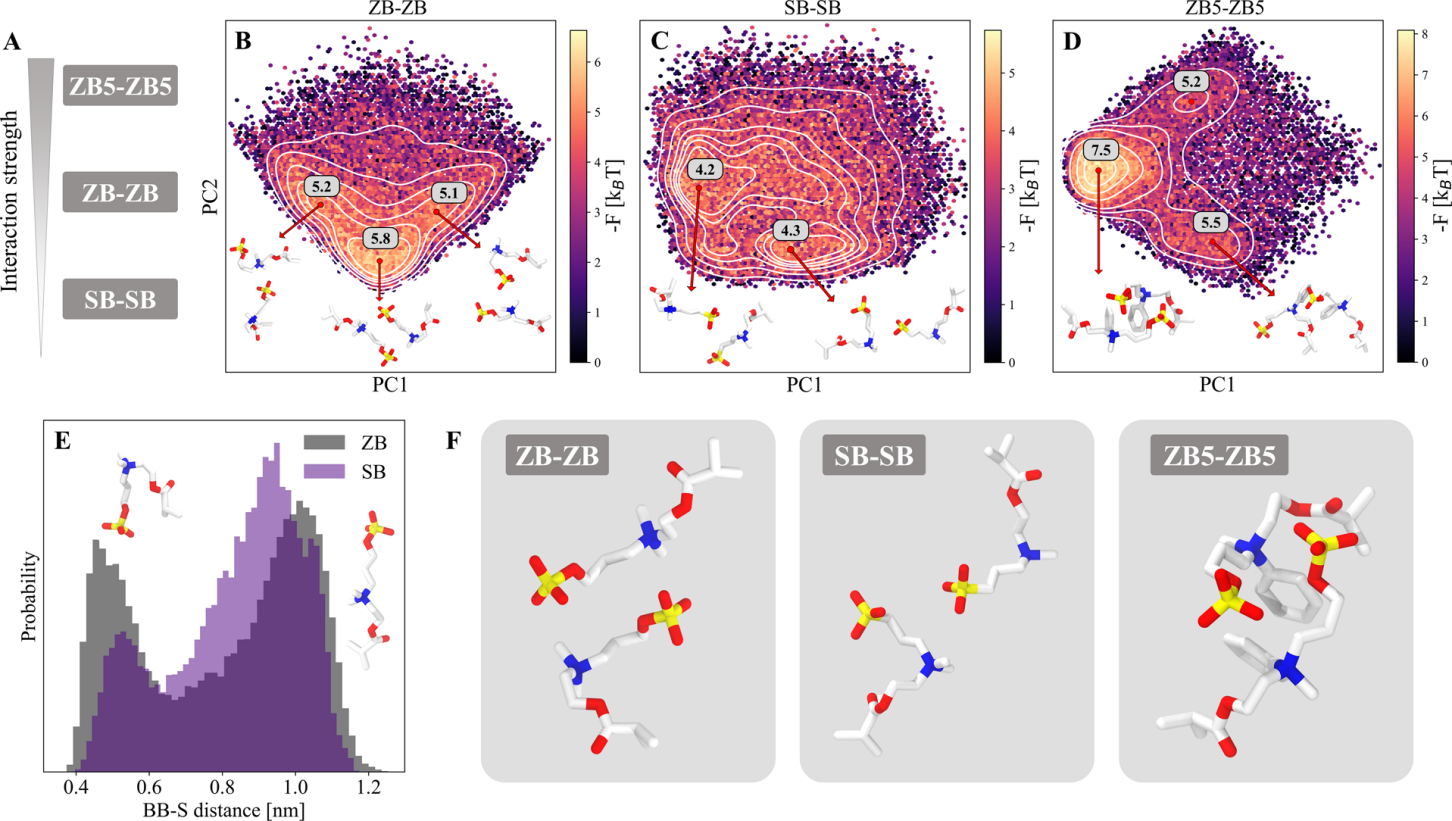
Conformational analysis of the Umbrella sampling trajectories provides molecular insights into homotypic interactions of different strengths (A). (B) For ZB–ZB interactions, the most stable conformation features both charged groups paired, indicating strong interactions. (C) In the case of SB–SB interactions, only one pairing occurs at a time, indicating overall weaker interactions. (D) ZB5 homotypic interactions resemble the ZB case but with additional interaction of the phenyl groups, increasing the stability of the conformation. (E) Probability distribution of the distance between backbone and sulfur atom. Unlike ZB, SB predominantly adopts an open conformation, reflecting the higher hydrophilicity of the sulfonate group compared with the sulfate group. (F) Representative examples for the most stable conformations of the different monomer interactions.

The results of this analysis indicate that electrostatic interactions are essential in all cases, with the negatively charged group frequently pairing with the nitrogen group of the opposing molecule. In the ZB–ZB case, three primary modes of interaction were observed (Figure 4B): the most prevalent mode involves a state where both charges are simultaneously paired, with the sulfate group of one molecule binding to the nitrogen group of another molecule. In the other two modes, this binding applies only to one of the two pairs. When compared to the structurally similar SB monomer, it is evident that the interaction modes differ significantly (Figure 4C). Although the sulfonate group still pairs with the nitrogen of the opposing molecule, the “double charge pairing” observed in the ZB case is absent. The two dominant modes in SB involve the binding of only one charge pair at a time. In contrast, in the strongly interacting ZB5 monomer, the “double charge pairing” reappears as the most stable configuration (Figure 4D). This configuration is further stabilized by the interaction of the phenyl groups, contributing to the increased interaction strength observed in the Umbrella sampling analysis.

Additionally, the analysis of the monomer trajectories provided further insights into the differences between the ZB and SB monomers. Both monomers exist in equilibrium between closed and open states, as reflected by the probability distribution of the backbone–sulfur distance (Figure 4E). For ZB, the two states have nearly equal probabilities, whereas in the case of SB, the open state is significantly more populated. These results indicate the higher hydrophilicity of the sulfonate group in SB compared to the sulfate group present in ZB,^50^ aligning with our previous findings.

Representative examples for the most stable configurations of the different monomer interactions are summarized in Figure 4F. It is important to note that the interaction strengths, also presented in Figures 1C and 4A, were quantified on the basis of not only these highly populated interaction modes but also all conformations explored during the simulations. Even though interactions appear to be primarily driven by the charged groups, a wide range of relative orientations was explored, accounting for interactions involving all parts of the molecules (Figure S9).

## Conclusions

In this study, we established a strategy to predict the phase separation behavior of zwitterionic polymers characterized by different functional groups. The interaction strength among different monomers, which was quantified through MD simulations, informed the macroscopic phase separation behavior of the corresponding homopolymers and enabled the design of stimulus-responsive behavior. We validated our approach by synthesizing a series of zwitterionic homopolymers that exhibited no phase separation, liquid–liquid phase separation, or liquid–gel phase separation.

Beyond their predictive power, the simulations provided valuable molecular insights into the distinct intermolecular interaction modes encoded by the different functional groups, rationalizing the drastically different phase separation behaviors observed with monomers that exhibit only minimal structural variations.

Overall, our findings provide crucial insights into the molecular grammar that dictates the phase separation behaviors of different zwitterionic polymers, offering an important design framework for the development of their materials.

## Methods

### Umbrella Sampling

To compute the PMF profiles for the monomer pairs, we performed Umbrella sampling simulations^45,46,51^ using a total of 28 windows, with COM distances equally spaced from 0.1 to 2.8 nm. For these values, we applied positional restraints using harmonic potentials with a force constant of 0.6 J mol^–1^ pm^–2^ in the *z* direction and added additional restraints of 25 J mol^–1^ pm^–2^ in perpendicular directions, reducing the size of the simulation box required (periodic boundary conditions). We chose the GAFF2 ^43^ force field with SPC/E water^44^ due to its accurate representation of the dipole moment and dielectric constant of water molecules. We added NaCl using the Joung–Cheatham model,^52^ corresponding to ∼300 mM, reflecting intermediate experimental conditions. Bonds involving hydrogen atoms were constrained using the LINCS algorithm,^53^ and a 1 fs time step was employed for integration. The cutoff for nonbonded potentials was set to 1.0 nm, and long-range electrostatics were treated using the particle-mesh Ewald method.^54^ Production runs were performed in the *NPT* ensemble applying the Nosé–Hoover thermostat^55,56^ (300 K, 1 ps) and a Parrinello–Rahman barostat^57^ (1 bar, 5 ps). The simulations were carried out using GROMACS 2021.4 ^58^ for 250 ns per window, and the first 50 ns were used for equilibration. To convert the obtained histograms into a PMF, we employed the WHAM analysis method^46,59^ together with the Bayesian bootstrap method for creating an ensemble of PMF profiles, finally leading to an error estimate. The interaction free energy was estimated as the difference between the minimum in the PMF (COM distance ≥0.4 nm) and the average value in a reference window (1.75–2.25 nm).

To prepare a system, two hydrogen atoms were added to the monomer backbone, leading to saturated bonds, and an initial monomer geometry was obtained from the LigParGen server,^60–62^ which was then optimized at the HF/6-31G* level in vacuum, followed by a calculation of the electrostatic potential (ESP) using the Gaussian09 software.^63^ The atomic charges were determined using the RESP method implemented in AmberTools,^64^ respecting the internal symmetries of the molecule. After solubilizing the system in a box of dimensions 26 × 26 × 70 A^3^ (∼4000 atoms) and the addition of salt, the initial configurations for the Umbrella sampling were generated by steered MD starting from 2.8 nm COM distance and ending at 0.1 nm (1 ns, 10 J mol^–1^ pm^–2^). This was followed by an energy minimization with a force tolerance of 1000 J mol^–1^ pm^–1^, leading to the initial configurations for the production runs.

### Reweighting Sampled Configurations

For conformational analysis of a monomer interaction, six umbrella windows covering the main well in the PMF profile (Figure S10) were analyzed (ZB: 0.5–1.0, SB: 0.7–1.2, ZB5: 0.4–0.9 nm) with 5000 frames each, leading to a total of 30,000 conformations per interaction. The trajectories were analyzed based on the coordinates of the carbonyl carbon, nitrogen, and sulfur atoms. We performed a principal component analysis on all combinations of interatomic distances as variables^65^ and used Zwanzig’s expression^66^ to assign weights *w* to each frame *j* from the umbrella window *i*:

$$w_{j}\propto \exp\left(\frac{-F_{i}+V_{z}(z_{j})+V_{r}(r_{j})}{k_{B}T}\right)$$ 1

where *F*_*i*_ is the alignment constant of window *i*, computed by iteratively solving the WHAM equations,^46^ and *V*_*z*_ and *V*_r_ are the values of the harmonic potentials in the pulling and orthogonal directions acting on configuration *j*. Frames with biasing potentials above 4 *k*_B_ *T* were excluded from the analysis. This leads to a representation of the equilibrium probability distribution in the principal component space, which can be converted into a free energy surface.^67^ To identify free energy minimum coordinates in the principal component space, we smoothed the free energy surface using kernel density estimation, followed by local minimization. The free energy values of the identified minima were determined by local averaging within a distance cutoff of 0.1 in the (PC1, PC2) space. Finally, sampling frames close to these minima gave insight into the most relevant conformations.

### Synthesis of Polymers and Characterization of Phase Separation

All polymers were synthesized via RAFT polymerization using 4,4′-azobis­(4-cyanovaleric acid) (ACVA) as an initiator and 4-cyano-4-(phenylcarbonothioylthio)­pentanoic acid (CPA) as a RAFT agent according to previously reported protocols with slight modifications.^15^ The monomer concentration was set to around 10 wt %, and the initiator to CPA molar ratio was set to 1/3. The targeted degree of polymerization (DP) was fixed to 200, where different comonomer ratios were varied, as reported in Table S3. As an example, polyZB was synthesized by dissolving 3.3 mg (11.2 μmol) of ACVA, 10 mg (35.8 μmol) of CPA, and 2.1 g (7.1 mmol) of ZB in 20 mL of solvent mixture consisting of ethanol/3 M NaCl acetic buffer (20/80, v/v, pH = 4.5) and poured in a 25 mL septum-sealed round-bottom flask equipped with a magnetic stirrer. The mixture was purged for 10 min by bubbling nitrogen and then placed in a preheated oil bath at 65 °C under magnetic stirring. The polymerization was carried out for 24 h. The reaction was stopped by cooling to room temperature and exposure to air. The reaction mixture was dialyzed against 1 or 3 M NaCl solution for 3 days with dialysis tubing (Spectra/Por, molecular weight cutoff (MWCO) = 3.5 kDa) by frequently changing the aqueous solution. The final product was then filtered using Whatman Syringe Filters (PP Filter Membrane, pore size = 0.45 μm). The final polymer concentration in the saline solution was evaluated via gravimetry. For the polymerization of polyZB5 and the copolymerization of ZB5 and SB, total monomer concentration was kept at 5 wt % in a solvent mixture consisting of ethanol/3 M NaCl acetic buffer (11/89, v/v, pH = 4.5). An undissolved gel was formed after polymerization of polyZB5, and the gel was taken out for photography purposes without purification (Figure 1C). For the copolymerization, the purification procedure was conducted in the same manner as the other polymers using 3 or 4 M NaCl solution as the dialysis medium. Polymerization conversion was evaluated by taking an aliquot of the reaction mixture after the reaction, which was dried under nitrogen and dissolved again in 3 M (4 M for p­(160ZB5-*co*-40SB)) NaCl D_2_O for ^1^H NMR.

The cloud point temperature *T*_cp_ of the polymers was determined using dynamic light scattering (DLS) at a constant polymer concentration of 0.25 mg/mL and NaCl concentration of 150 mM. To quantify the maximum salt concentration for phase separation *C*_s,max_, we monitored phase separation under an epi-fluorescence microscope at ambient temperature and a total polymer concentration of also 0.25 mg/mL. All solutions were prepared in deionized water; pH effect was not explored in this study. More details regarding monomer synthesis and characterization of phase separation are reported in the Supporting Information.

## Supplementary Material



## Notes

Details regarding materials, monomer and polymer synthesis and characterization, as well as interaction free energy values and potential of mean force profiles (PDF)
