# Supplementary material for: Rational Design of Zwitterionic Polymers with Tunable Phase Separation Propensity
Source: Macromolecules. 2025 Oct 8;58(20):11192–9. doi: 10.1021/acs.macromol.5c01394 (PMC12573804; doi:10.1021/acs.macromol.5c01394)
Supplement: Supplementary file 1 [file ma5c01394_si_001.pdf]

Supplementary Information: Rational design of zwitterionic polymers with tunable phase separation propensity

Timo N. Schneider,† Suiying Ye,† Nicola Carrara,† Umberto Capasso Palmiero,† Matteo Salvalaglio,‡ and Paolo Arosio*,†

†*Department of Chemistry and Applied Biosciences ETH Zurich, Vladimir Prelog Weg 1, 8093, Zurich, Switzerland*

‡*Department of Chemical Engineering, University College London, London WC1E 6BT, U.K.*

E-mail: paolo.arosio@chem.ethz.ch

Materials

1,3,2-Dioxathian-2,2-dioxide (TMS, 98%, MW = 138.14, Sigma Aldrich), tert-butyl bromoacetate (98%, MW = 195.05, Sigma Aldrich), 1,3,2-Dioxathiolane-2,2-dioxide (TMS2, 98%, MW = 124.12, Sigma Aldrich), 2-(Dimethylamino)ethyl methacrylate (DMAEMA, 98%, MW = 157.21, Sigma Aldrich), 2-(Diethylamino)ethyl methacrylate (DEAEMA, 99%, MW = 185.26, Sigma Aldrich), 2-N-Morpholinoethyl methacrylate (MEMA, 95%, MW = 199.25, Sigma Aldrich), N-(3-sulfopropyl)-N-methacroyloxyethyl- N,N-dimethylammonium betaine (SB, ≥98.0%, MW = 279.35, Merck), 4,4'-azobis(4-cyanovaleric acid) (ACVA, ≥98%, MW = 280.28, Sigma Aldrich), 4-cyano-4-(phenylcarbonothioylthio)pentanoic acid (CPA, ≥97%, MW = 279.38, Sigma Aldrich), 2-Methacryloyloxyethyl phosphorylcholine (MPC, 97%, MW = 295.27, Sigma Aldrich), ethanol (≥99.8%, MW = 46.07, Sigma Aldrich), acetonitrile (ACN, 99.99%, MW = 41.05, Fisher Chemicals), acetonitrile (99.9%, extra dry over molecular sieve, MW = 41.05, Fisher Scientific), diethyl ether (99.5%, MW = 74.12, Sigma Aldrich), sodium acetate (NaOAc, ≥99.0%, MW=82.03, VWR), acetic acid (AcOH, ≥99%, MW=60.05, Sigma Aldrich), sodium chloride (NaCl, ≥99.5%, MW = 58.44, Sigma Aldrich), sodium perchlorate (NaClO4, ≥98%, MW = 122.44, Sigma Aldrich), sodium bicarbonate (NaHCO3, >99.7% crytals, MW = 84, Fisher Scientific), sodium sulfate anhydrous (Na2SO4, ACS reagent, ≥99.0% anhydrous, granular), 2-(methylphenylamino)ethanol (98%, MW = 151.20, Sigma Aldrich), methacryloyl chloride (97%, MW = 104.53 Sigma-Aldrich), hydroquinone (>99.0%, MW = 110.11, Tokyo Chemical Industry), chloroform (99.9%, extra dry over molecular sieve, MW = 119.37, Fisher Scientific), triethylamine (TEA, ≥99.5%, MW = 101.19, Sigma-Aldrich) methanol-d4 (99.8%, MW = 36.06, Sigma Aldrich), deuterium oxide (D_2O, 100%, MW = 20.02, Sigma Aldrich), chloroform-d (CDCl3, 99.8 atom % D, Fisher Scientific), albumin–fluorescein isothiocyanate conjugate (BSA-FITC, Sigma Aldrich), rhodamine B (RhB, 99+%, Acros) were used as received. All solvents were of analytical grade purity and used without further treatment.

Monomer synthesis

A protected CB monomer was synthesized via addition of tert-butyl bromoacetate with DMAEMA. Briefly, 11 mL of DMAEMA and 19 mL of tert-butyl bromoacetate were dissolved in 60 mL of acetonitrile in a round-bottom flask with a magnetic stirrer. The mixture was left to react at 50 °C under a protective atmosphere of nitrogen for 2 days and then evaporated under vacuum. The final product was washed three times with diethyl ether, dried under vacuum, and characterized via 1H NMR in D_2O.

The ZB monomer was synthesized via an addition reaction according to a procedure previously reported.[1] Briefly, 10 g of DMAEMA and 8 g of TMS were respectively dissolved in 10 mL and 70 mL of acetonitrile at ambient temperature. The solutions were added to a 250 mL septum-sealed round bottom flask equipped with a magnetic stirrer and the reaction was carried out at 50 °C for 3 days. At the end of the reaction, the monomer was precipitated by cooling the mixture to ambient temperature and by adding acetone. After filtration and further washing with acetone, the solid was dried under vacuum to remove all the residual acetone and characterized via 1H NMR in D_2O. The ZB2 monomer was synthesized via an addition reaction, where 5.56 g of DMAEMA and 4 g of TMS2 were respectively dissolved in 5 mL and 35 mL of acetonitrile at ambient temperature. The ZB3 monomer was synthesized via an addition reaction, where 5.89 g of DEAEMA and 4 g of TMS were respectively dissolved in 5 mL and 35 mL of acetonitrile at ambient temperature. The ZB4 monomer was synthesized via an addition reaction, where 6.34 g of MEMA and 4 g of TMS2 were respectively dissolved in 5 mL and 35 mL of acetonitrile at ambient temperature. The rest of the procedure is the same as the one used to produce ZB monomer.

Synthesis of 3-((2-(methacryloyloxy)ethyl)(methyl)(phenyl)ammonio)propyl sulfate (ZB5) was carried out in 2 steps. First, 2-(methylphenylamino)ethanol (6.11 g) were dissolved in 15.9 g chloroform in a round bottom flask, followed by loading of 7.89 mL TEA. The flask was then cooled in an ice bath and bubble with N2 for 10 min. Methacryloyl chloride (4.83 g) in 15.9 g chloroform was added dropwise to the reaction flask under ice temperature and N2

environment with stirring over 20 min. The ice bath was then removed and let the reaction mixture return to room temperature, followed by stirring overnight. The reaction mixture was washed with 50 mL saturated $NaHCO_3$ solution for 3 times and 50 mL saturated NaCl solution for 2 times. The organic phase was collected, dried with Na_2SO_4, and concentrated using a rotary evaporator. The product of the first step, 2-(methyl(phenyl)amino)ethyl methacrylate, was purified by column chromatography using ethyl acetate/hexane (v/v, 1/40) and characterized with 1H NMR in CDCl3. 2-(Methyl(phenyl)amino)ethyl methacrylate (1.07 g), TMS (0.68 g), and hydroquinone (22.5 mg) were dissolved in 5 mL dry acetonitrile. The mixture was heated at 90 °C for 2 days with stirring. After cooling down the reaction mixture, 70 mL acetone was added into the reaction flask to precipitate the zwitterionic product. The white solid was collected and re-dissolved in deionized water, followed by 3 washing cycles with diethyl ether. The organic solvent was removed under heat and the product as white powder was collected through freeze-drying. The total yield of the two steps was determined to be about 48%. The final product was characterized with 1H NMR and 13C NMR in D_2O.

1H NMR of 2-(Methyl(phenyl)amino)ethyl methacrylate (CDCl3, 300 MHz), δ (ppm) = 7.13 (t, aromatic, 2H), 6.65 (m, aromatic, 3H), 5.97 (s, CHH=C(CH3)-, 1H), 5.44 (s, CHH=C(CH3)-, 1H), 4.22 (t, -OCH2CH2N(CH3)-, 2H), 3.54 (t, -OCH2CH2N(CH3)-, 2H), 2.88 (s, -OCH2CH2N(CH3)C6H5, 3H), 1.81 (s, CHH=C(CH3)-, 3H). 1H NMR of PhZB (D2O, 300 MHz), δ (ppm) = 7.84 – 7.60 (m, aromatic, 5H), 5.89 (s, CHH=C(CH3)-, 1H), 5.65 (s, CHH=C(CH3)-,1H), 4.70-4.50 (m, -OCH2CH2N+(CH3)(C6H5)-, 2H), 4.40-3.93 (m, -OCH2CH2N+(CH3)(C6H5)-, -CH2N+(CH3)(C6H5)CHHCH2CH2-, -CH2CH2OSO3, 6H), 3.74 (s, -OCH2CH2N+(CH3)(C6H5)-, 3H), 2.22-2.06 (m, -N+(CH3)(C6H5)CH2CHHCH2OSO3-, 1H), 1.77 (s, CH2=C(CH3)-, 3H), 1.74-1.60 (m, -NCH2CHHCH2OSO3- , 1H). 13C NMR of PhZB (D2O, 75 MHz), δ (ppm) = 168.17, 140.97, 134.65, 130.83, 127.81, 121.37, 116.37, 67.81, 66.62, 65.13, 58.36, 47.68, 22.63, 17.05.

Polymer characterization

Size exclusion chromatography. The number-averaged molecular weight (Mn) and dispersity (Đ) of all the copolymers were evaluated via size exclusion chromatography. Samples were dissolved at 4 mg mL^{-1} in 100 mM $NaClO_4$/ acetonitrile (80/20 v/v) solution and filtered through a 0.45 μm pore-size nylon membrane. The separation was performed on a high-performance liquid chromatography system at a flow rate of 1 mL min^{-1} at room temperature with one guard and two Suprema columns (particle size 10 mm and pore sizes 100 and 1000 Å, Polymer Standards Service). The values are reported in Table S3 and are relative to polyethylene glycol standards (ReadyCal-Kit PEO/PEG, Mp = 238-969 000 Da, Polymer Standards Service).

Nuclear magnetic resonance. The polymerization conversion (X) and molar fraction of ZB and its derivatives of all copolymers (Table S3) were evaluated via nuclear magnetic resonance (1 H-NMR) as described below. An aliquot of all the reaction mixtures was withdrawn before and after the reaction completion. The samples were dried under nitrogen, dissolved in either 3 M NaCl D_2O for all ZB-based copolymers and analyzed on a 400 MHz NMR spectrometer (Bruker).

Cloud point measurement. The cloud point temperature, Tcp, was measured via dynamic light scattering (DLS) (Zetasizer Nano ZS, Malvern Instruments) at a scattering angle of 173°. 200 μL of the sample at 0.25 mg mL^{-1} polymer concentration and 150 mM NaCl were left to equilibrate at 65°C for 20 minutes before analysis. The scattered light and droplet size were then measured from 65°C to 5°C with a temperature step of 1°C and an equilibration time of 10 min. The cloud point was defined as the temperature where the Z-average hydrodynamic radius first increases, as shown in Figure S1.

Maximum salt concentration measurement. Polymer phase separation was monitored under *epi*-fluorescence microscope (Eclipse Ti-E, Nikon) using a 60x oil objective (CFI Plan Apo Lambda, Nikon) at room temperature. The polymer stock solution was added to different buffers to achieve desired salt concentrations in a 384-well plate pre-treated with 1%

BSA. Images were taken after 24 h of incubation under room temperature. The maximum salt concentration, $C_{\mathrm{s,max}}$, was evaluated as the maximum concentration at which coacervates were still visible within the resolution of the microscope.

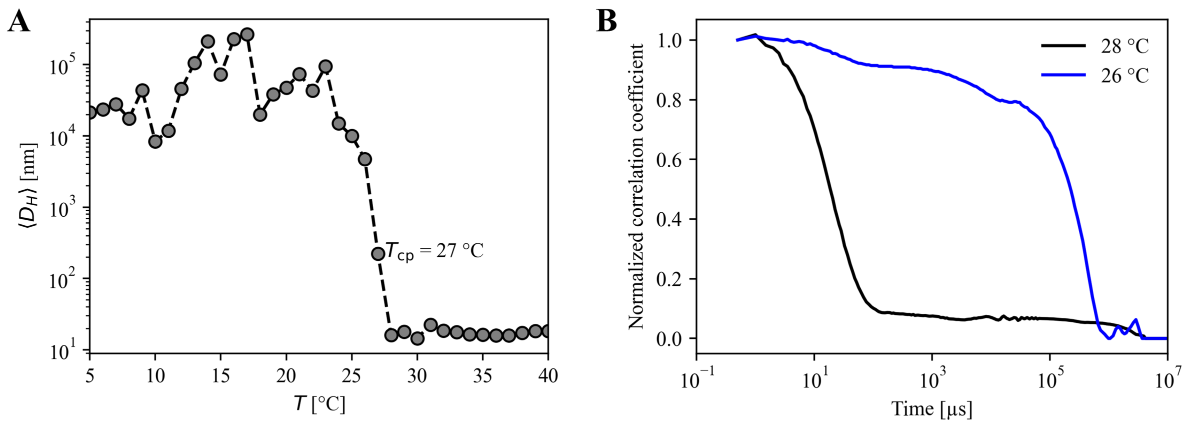

Fig. S1: Example for identifying the cloud point temperature using dynamic light scattering for the ZB2-SB copolymer ($f_{M1} = 56$ %). (A) Z-average hydrodynamic diameter at decreasing temperature. (B) Autocorrelation functions above (T = 28 °C) and below (T = 26 °C) the cloud point temperature.

Tab. S1: Homotypic interaction free energies of all monomers quantified with Umbrella sampling simulations.

**Monomer**	**Molecular structure**	$F_{\mathrm{min}}[k_{\mathrm{B}}T]$	Synthesized
M1	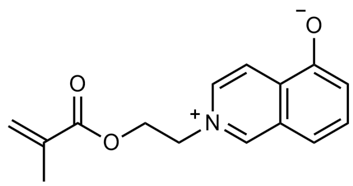	4.60 ± 0.07	No
M2	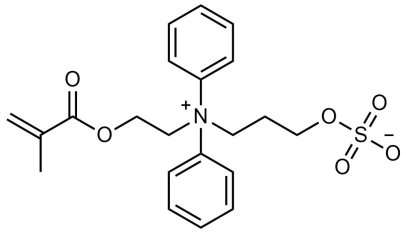	3.57 ± 0.12	No
M3	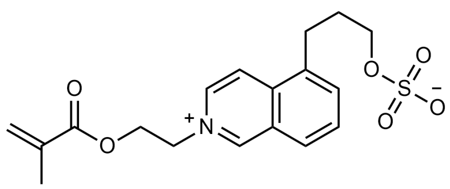	3.53 ± 0.12	No
M4	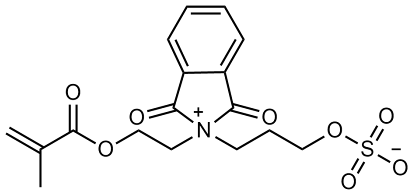	3.45 ± 0.19	No

Tab. S1 (continued)

**Monomer**	**Molecular structure**	$F_{\mathrm{min}}[k_{\mathrm{B}}T]$	Synthesized
M5	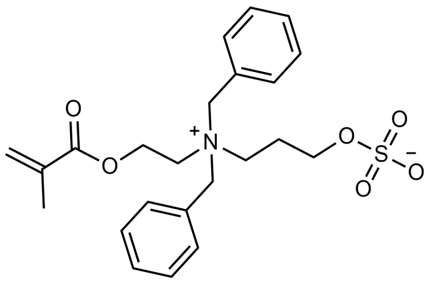	2.90 ± 0.08	No
ZB5	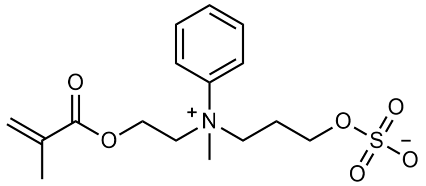	2.77 ± 0.23	Yes
M6	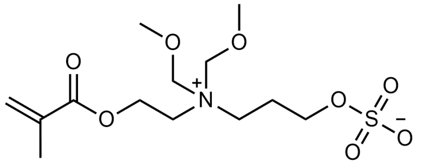	2.43 ± 0.14	No
M7	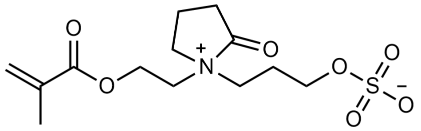	1.97 ± 0.12	No

Tab. S1 (continued)

**Monomer**	**Molecular structure**	$F_{\min}[k_{\mathrm{B}}T]$	Synthesized
M8	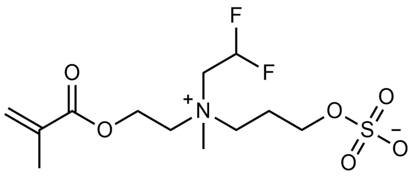	1.70 ± 0.04	No
M9	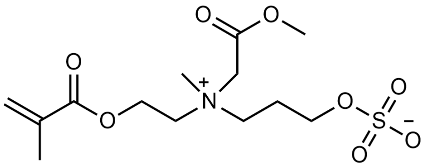	1.54 ± 0.06	No
M10	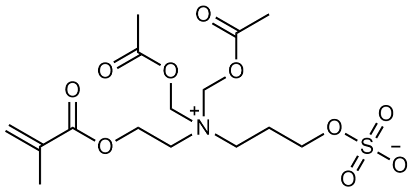	1.50 ± 0.04	No
M11	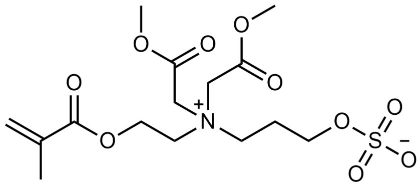	1.43 ± 0.11	No
ZB4	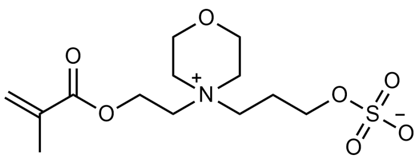	1.31 ± 0.06	Yes

Tab. S1 (continued)

**Monomer**	**Molecular structure**	$F_{\min}[k_{\mathrm{B}}T]$	Synthesized
ZB2	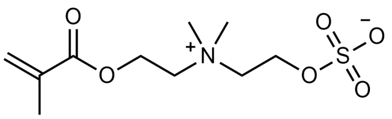	1.25 ± 0.04	Yes
M12	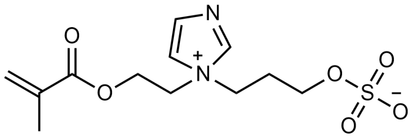	1.25 ± 0.03	No
M13	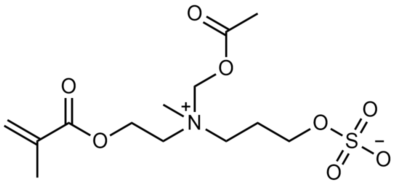	1.19 ± 0.05	No
M14	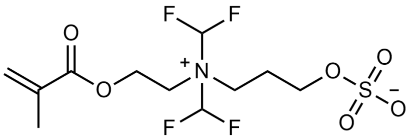	1.15 ± 0.06	No
ZB	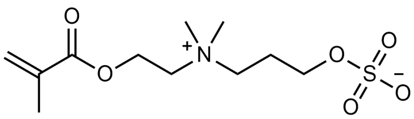	1.03 ± 0.04	Yes

Tab. S1 (continued)

**Monomer**	**Molecular structure**	$F_{\mathrm{min}}[k_{\mathrm{B}}T]$	Synthesized
M15	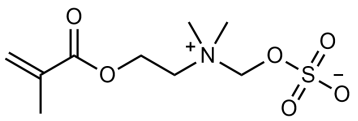	1.03 ± 0.04	No
ZB3	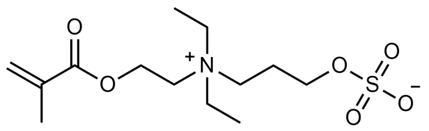	0.94 ± 0.05	Yes
M16	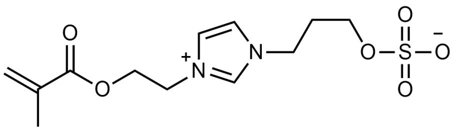	0.81 ± 0.02	No
M17	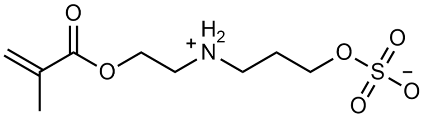	0.74 ± 0.03	No
M18	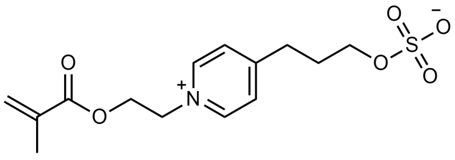	0.74 ± 0.02	No

Tab. S1 (continued)

**Monomer**	**Molecular structure**	$F_{\min}[k_\mathrm{B}T]$	Synthesized
M19	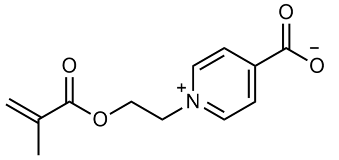	0.63 ± 0.04	No
MPC	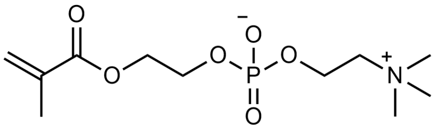	0.62 ± 0.03	Yes
SB	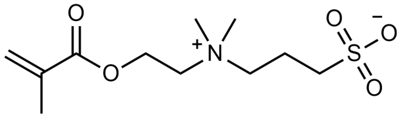	0.47 ± 0.04	Yes
CB	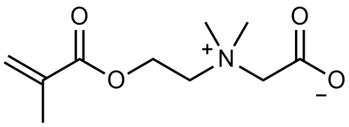	0.12 ± 0.03	Yes

Tab. S2: Heterotypic interaction free energies of experimentally synthesized cases, assessed through Umbrella sampling simulations.

**Monomer 1**	**Monomer 2**	$F_{\mathrm{min}}[k_{\mathrm{B}}T]$
ZB	SB	0.52 ± 0.05
ZB2	SB	0.77 ± 0.04
ZB3	SB	0.86 ± 0.04
ZB4	SB	0.73 ± 0.03
ZB5	SB	0.69 ± 0.02
ZB	CB	0.53 ± 0.02
ZB	MPC	0.72 ± 0.04

Tab. S3: Raw data for all synthesized polymers.

**Mon 1**	**Mon 2**	M_n [Da]	$Đ_{GPC}$ [-]	f_{M1} [%]	X [%]	T_{cp} [°C]	$C_{s,max}$
ZB	-	63,600	1.1	100	-	≥65	465 ± 10
ZB2	-	52,600	1.1	100	99	≥65	740 ± 10
ZB3	-	60,800	1.1	100	99	33 ± 1	230 ± 10
ZB4	-	59,500	1.1	100	99	≥65	605 ± 10
ZB5	-	-	-	100	-	-	-
SB	-	55,000[1]	-	100	98	-	-
CB	-	54,200[1]	-	100	99	-	-
MPC	-	58,200[1]	-	100	98	-	-
ZB	SB	46,600	1.3	47	98	7 ± 1	95 ± 5
ZB	SB	53,900	1.1	63	-	24 ± 1	185 ± 5
ZB	SB	10,800	1.2	76	99	41 ± 1	305 ± 5
ZB	SB	29,400	1.1	85	99	60 ± 1	390 ± 10
ZB2	SB	27,500	1.2	45	99	8 ± 1	100 ± 10
ZB2	SB	33,600	1.1	56	99	27 ± 1	185 ± 10
ZB2	SB	32,300	1.1	73	99	48 ± 1	405 ± 10
ZB2	SB	37,200	1.1	88	99	≥65	550 ± 10
ZB3	SB	55,500	1.1	51	99	≤5	28 ± 10
ZB3	SB	81,200	1.2	65	98	≤5	67 ± 10
ZB3	SB	62,300	1.1	78	99	9 ± 1	120 ± 10
ZB3	SB	67,500	1.1	88	99	31 ± 1	195 ± 10
ZB4	SB	46,700	1.1	53	99	≤5	67 ± 10
ZB4	SB	64,000	1.1	64	99	23 ± 1	145 ± 10
ZB4	SB	60,00	1.1	77	99	44 ± 1	305 ± 10
ZB4	SB	100,000	1.2	88	99	58 ± 1	450 ± 10
ZB5	SB	59,400[1]	-	55	92	≥65	1440 ± 10
ZB5	SB	63,000[1]	-	67	95	≥65	2320 ± 10
ZB5	SB	64,900[1]	-	79	95	≥65	3680 ± 10
ZB	CB	53,200	1.3	55	98	≤5	0
ZB	CB	55,600	1.2	73	98	≤5	0
ZB	CB	61,200	1.2	85	99	15 ± 1	125 ± 10
ZB	CB	53,000	1.2	95	98	46 ± 1	295 ± 10
ZB	MPC	56,700	1.2	54	98	≤5	0
ZB	MPC	55,000	1.2	67	99	≤5	0
ZB	MPC	30,000	1.3	83	99	≤5	0
ZB	MPC	24,300	1.3	93	99	18 ± 1	120 ± 10
ZB	MPC	34,000	1.4	97	99	41 ± 1	270 ± 10

[1]Molecular weight calculated from NMR.

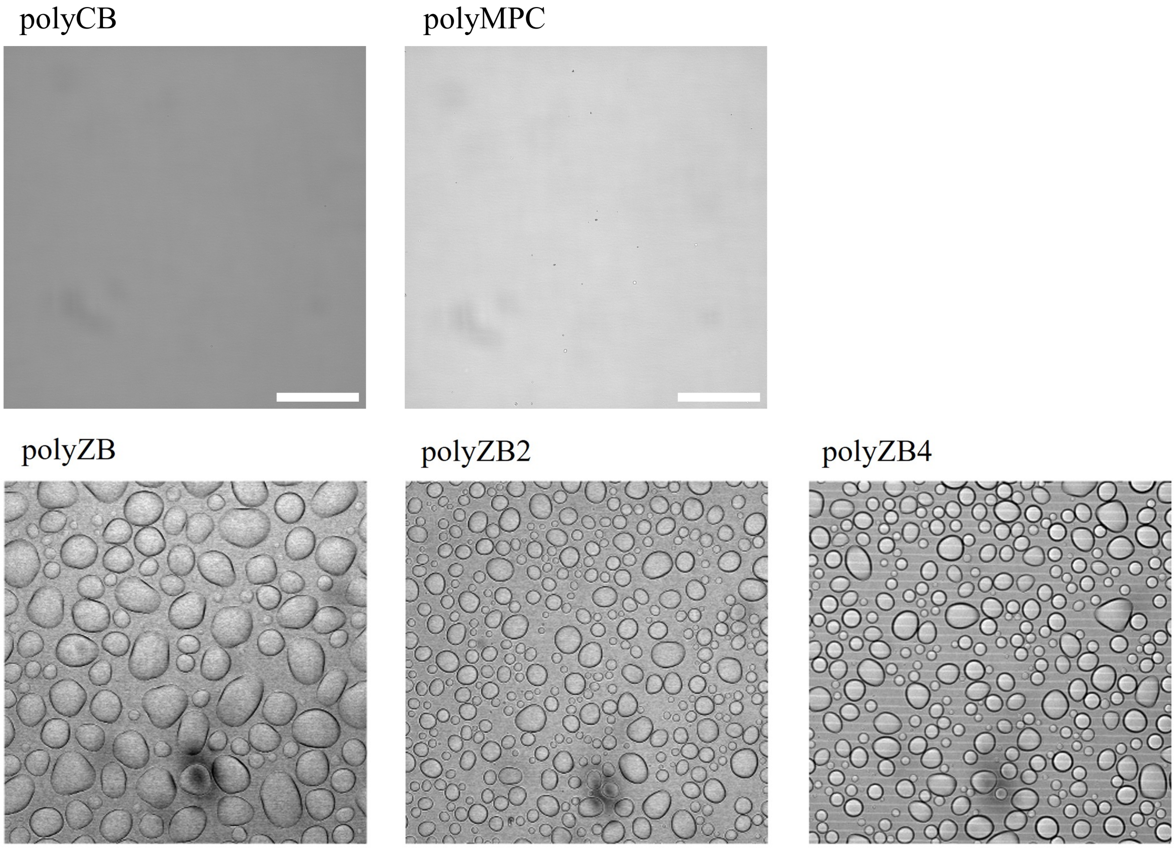

Fig. S2: Microscope images of polyCB, polyMPC, polyZB, polyZB2 and polyZB4 (0.25 mg/mL in 150 mM NaCl). The scale bar is 50 μm.

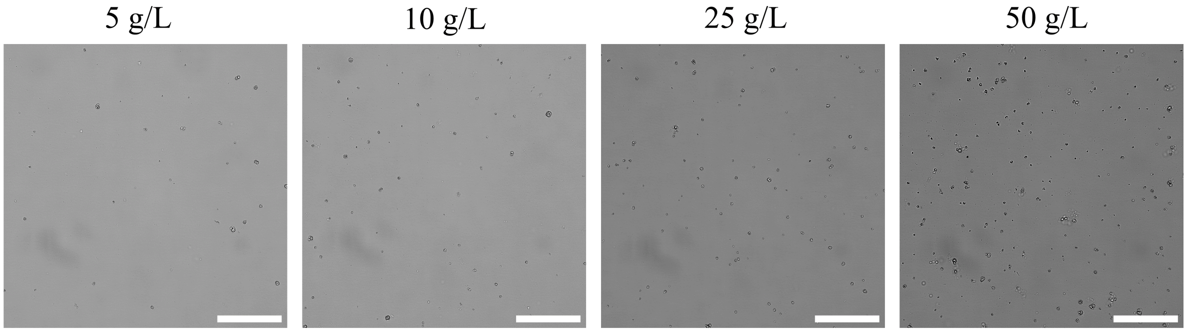

Fig. S3: Microscope images of polySB in 150 mM NaCl at different polymer concentrations. The scale bar is 50 μm.

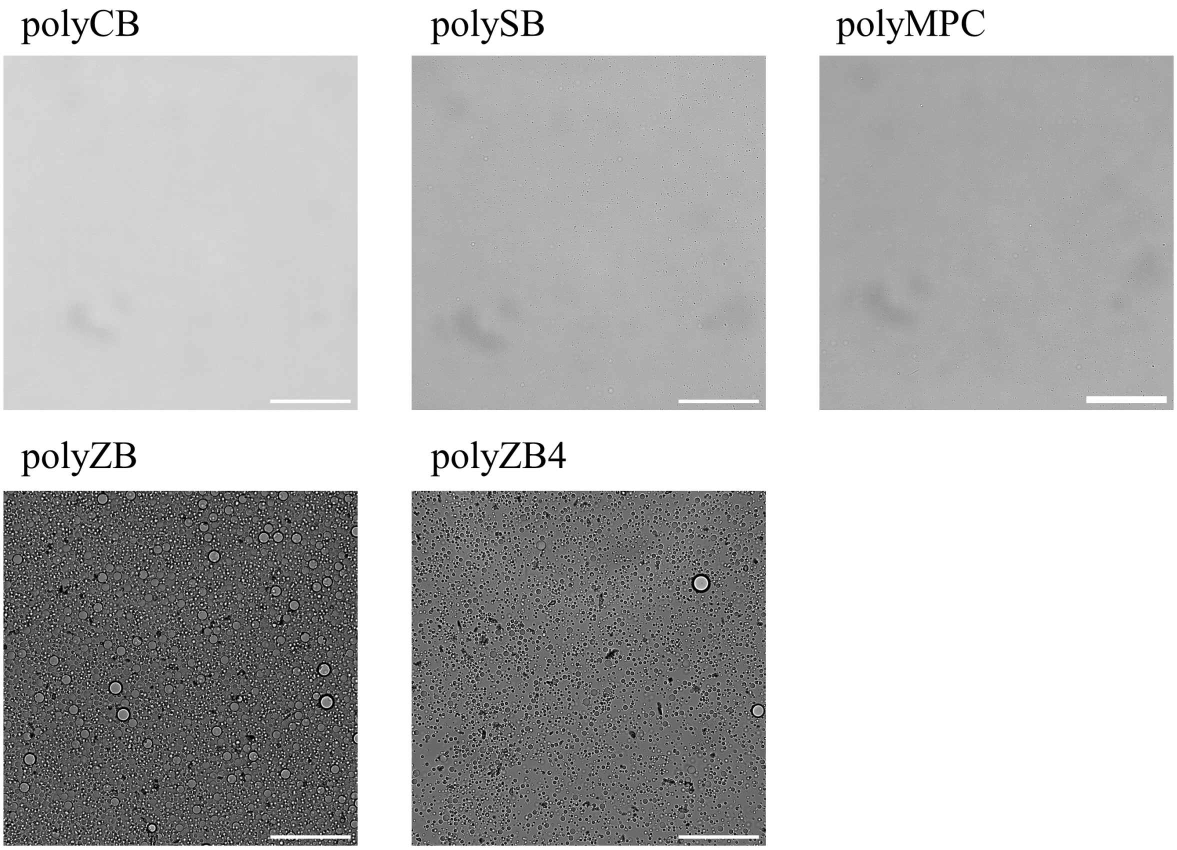

Fig. S4: Brightfield microscopy images of polyCB, polySB, polyMPC, polyZB, and polyZB4 in deionized water. Polymer concentrations were 0.25 mg/mL for polyCB, polySB, polyMPC, 0.18 mg/mL for polyZB, and 0.19 mg/mL for polyZB4. For polyZB and polyZB4, a solution of polymer at 0.25 mg/mL in high salt solution was prepared, followed by dialysis against deionized water overnight. For the non-phase separating polymers (polyCB, polySB, polyMPC), the polymers obtained in powder form after lyophilization were re-dissolved directly in deionized water. The non-spherical particles are impurities originating from the dialysis procedure. The scale bar is 50 μm.

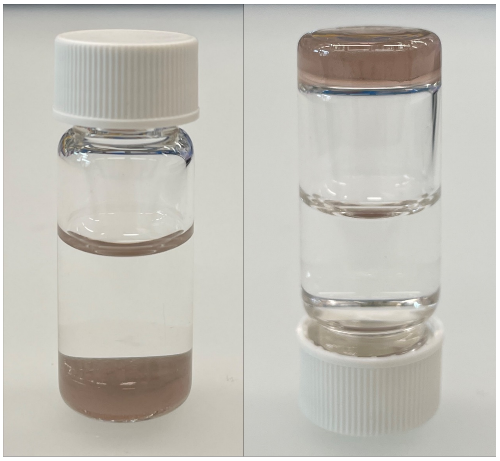

Fig. S5: polyZB5 in 3 M NaCl after 10 months of incubation. The slight color change compared to Figure 1C likely originates from reaction residues.

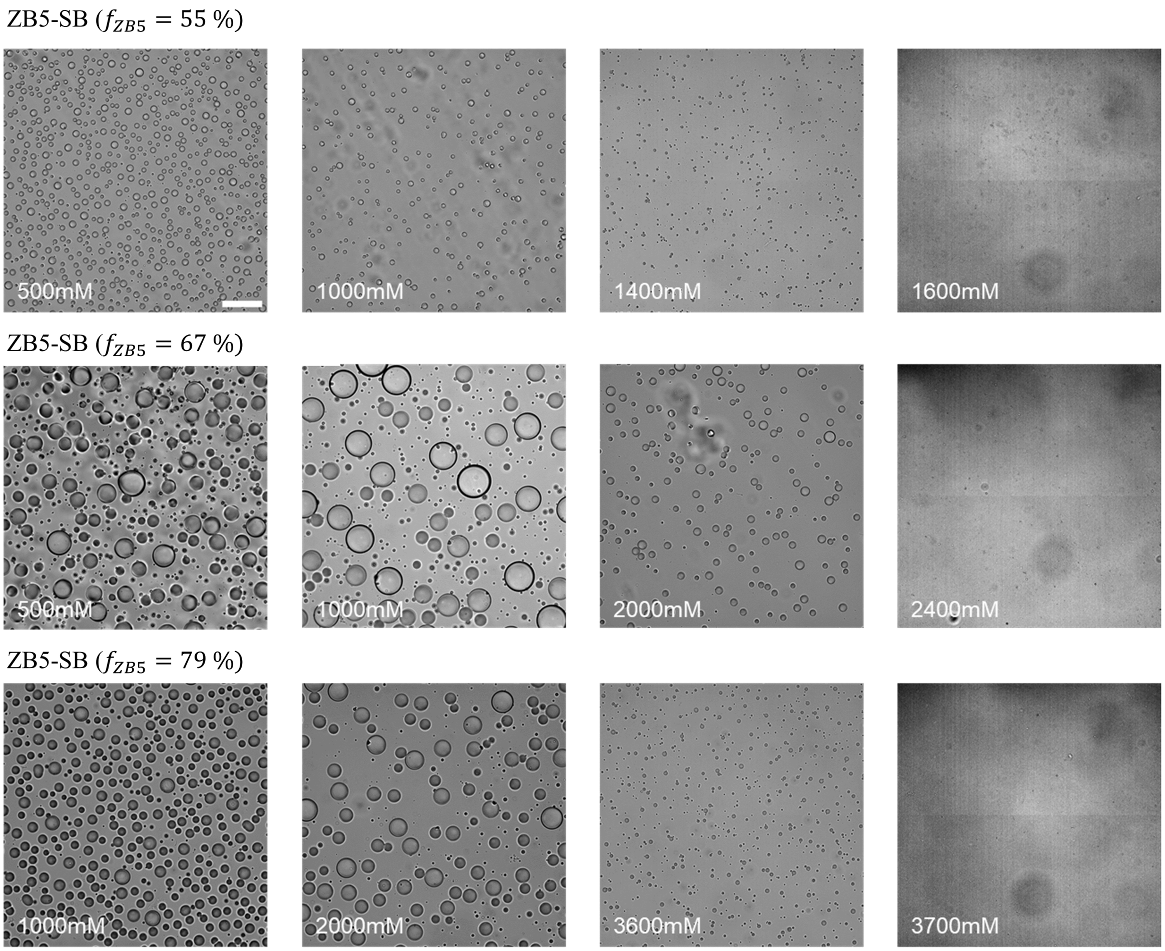

Fig. S6: Microscope images of the three ZB5-SB copolymers (0.25 mg/mL) at different salt concentrations. The scale bar is 100 μm.

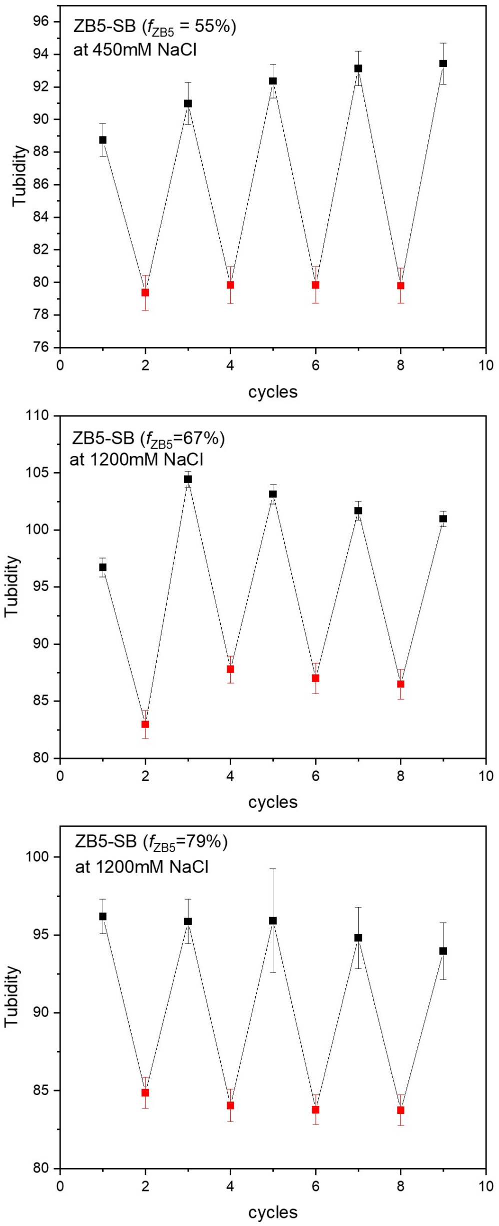

Fig. S7: Turbidity measurements of ZB5-SB copolymers under cycles of 25 °C (black) and 95 °C (red) at 0.25 mg/mL at different salt concentrations. The turbidity of 5 replicated samples was recorded after 10 min equilibration of each temperature using Prometheus Panta (nanotemper).

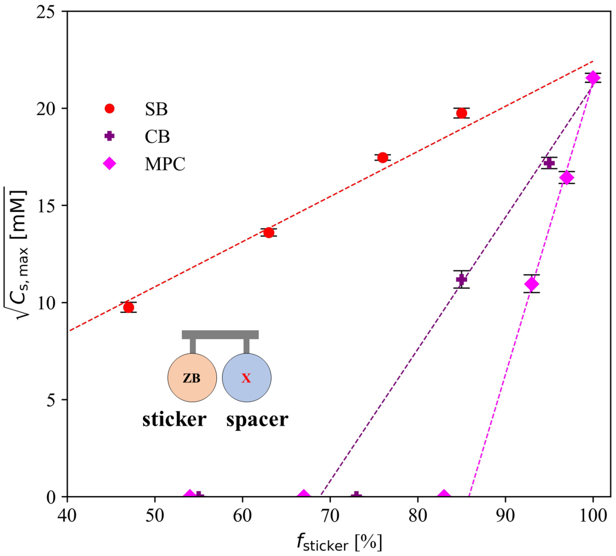

Fig. S8: Measured $\sqrt{C_{\mathrm{s,max}}}$ for different copolymers containing increasing fractions of ZB monomer.

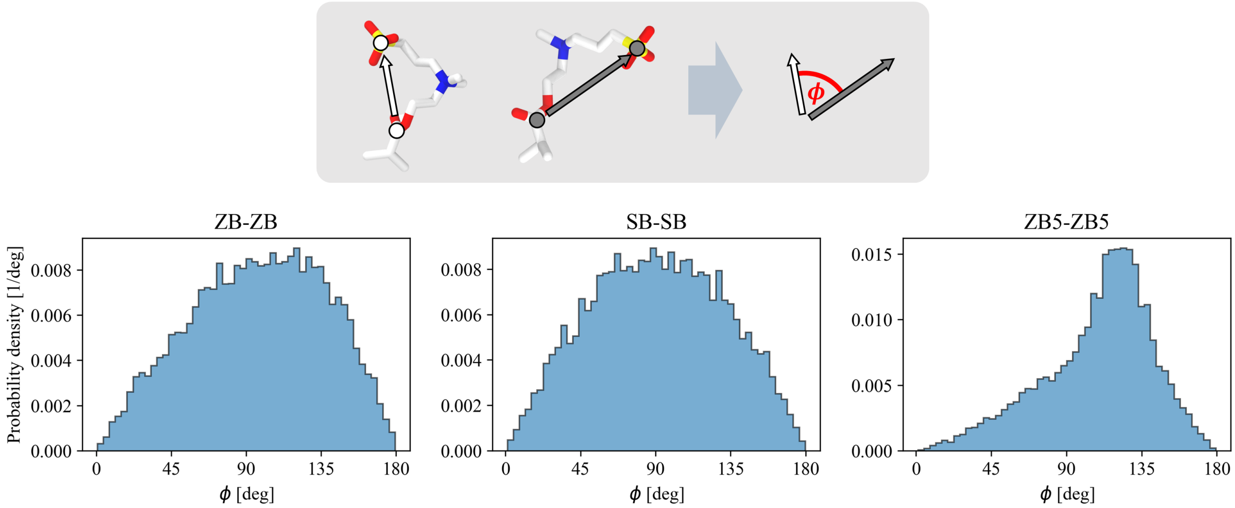

Fig. S9: Relative orientation of the monomer pairs analyzed in Figure 4B-D. The angle ϕ between the vectors defined by the carbonyl and sulfur atoms spans 0°-180° in all three cases, indicating that multiple interaction modes and orientations are explored. The first two distributions closely resemble a theoretical random distribution of angles,[2] while the third suggests that there is a clear preferential orientation, aligning with the high ZB5-ZB5 interaction strength. The probability density was calculated using the reweighting scheme described in the Methods section.

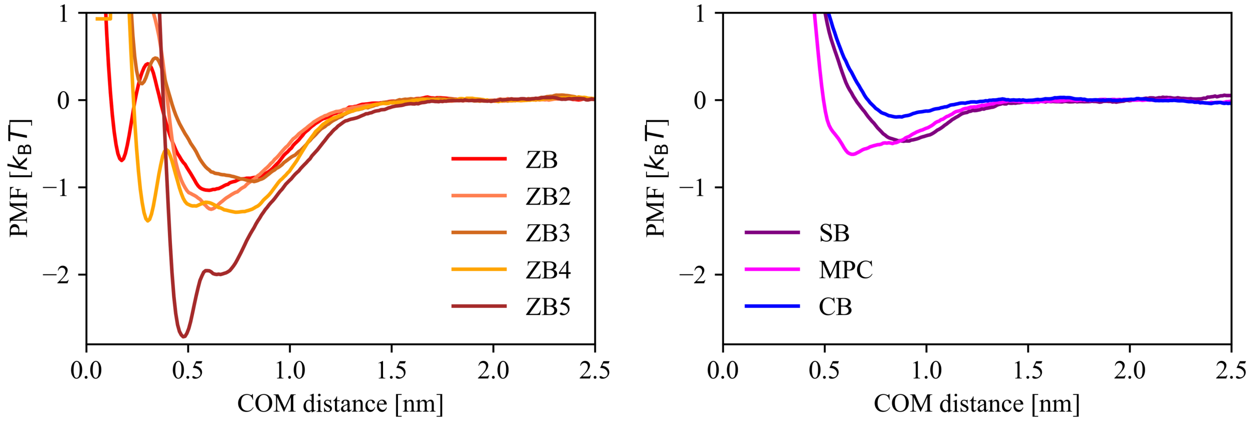

Fig. S10: Potential of mean force (PMF) profiles for the homotypic interactions of all synthesized zwitterionic monomers, obtained through Umbrella sampling. The profiles clearly show that the ZB variants (left) exhibit stronger attractive interactions compared to the spacer monomers (right).

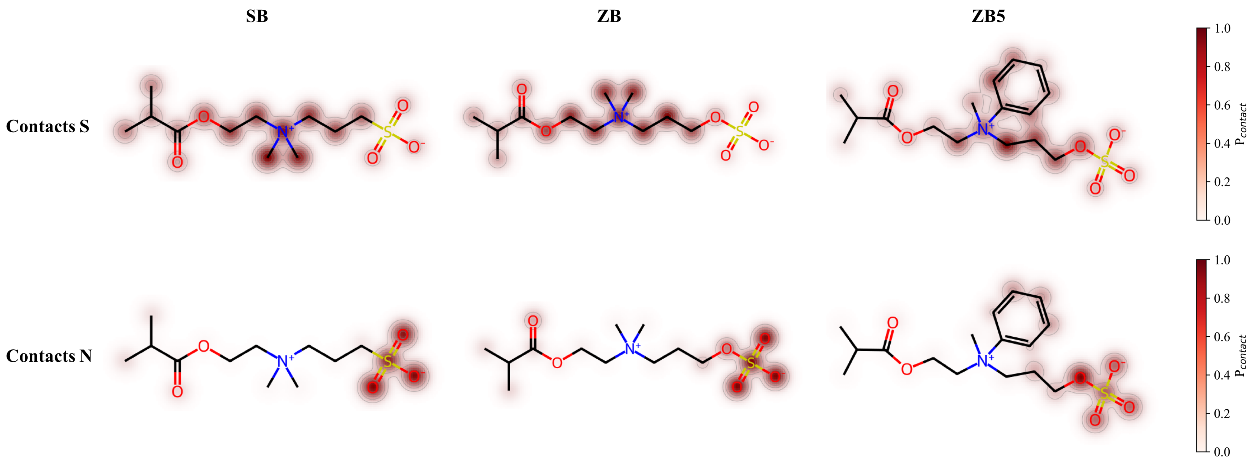

Fig. S11: Normalized intermolecular contact probabilities for the sulfur atom (top) and nitrogen atom (bottom) with all heavy atoms of the other molecule (cutoff: 0.5 nm) across three different monomers. In all cases, interactions with oppositely charged groups are predominantly observed.

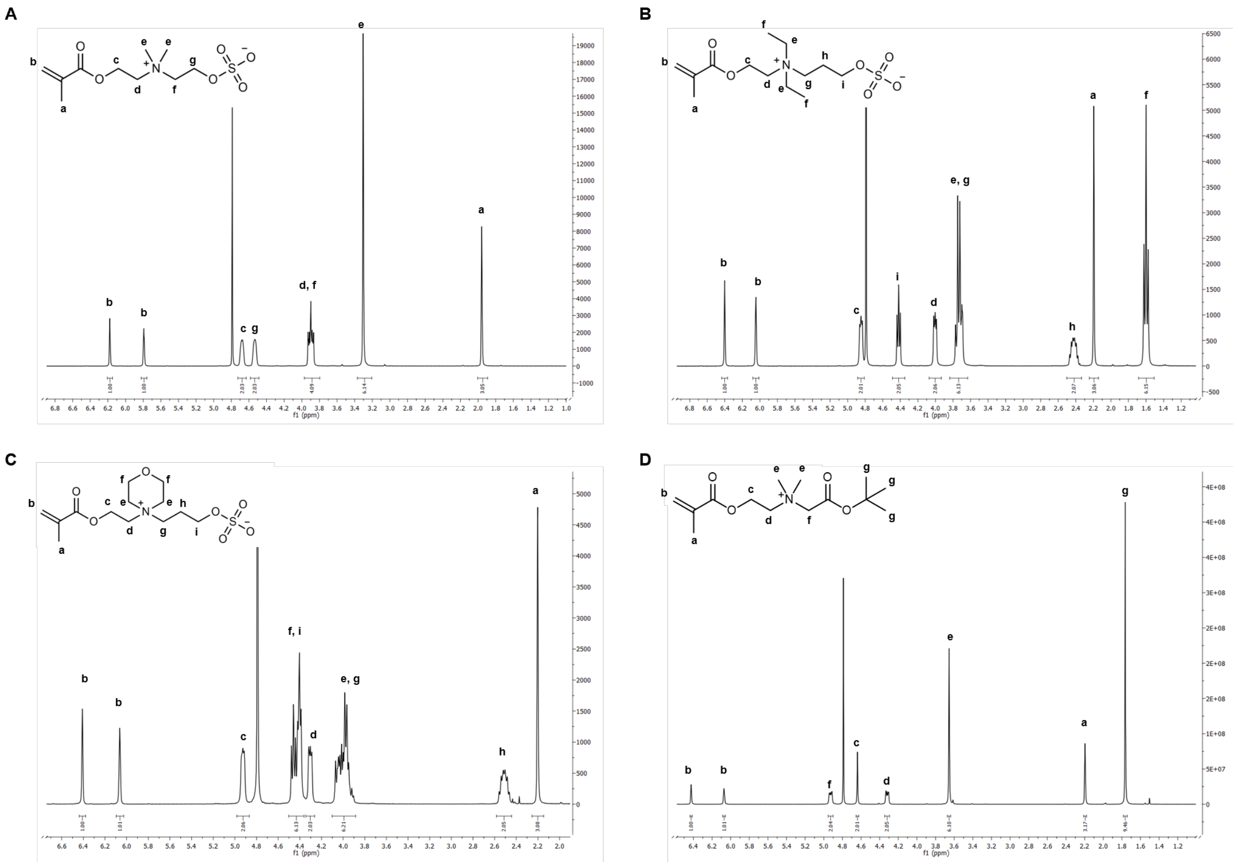

Fig. S12: NMR spectra of the monomers synthesized in this work.

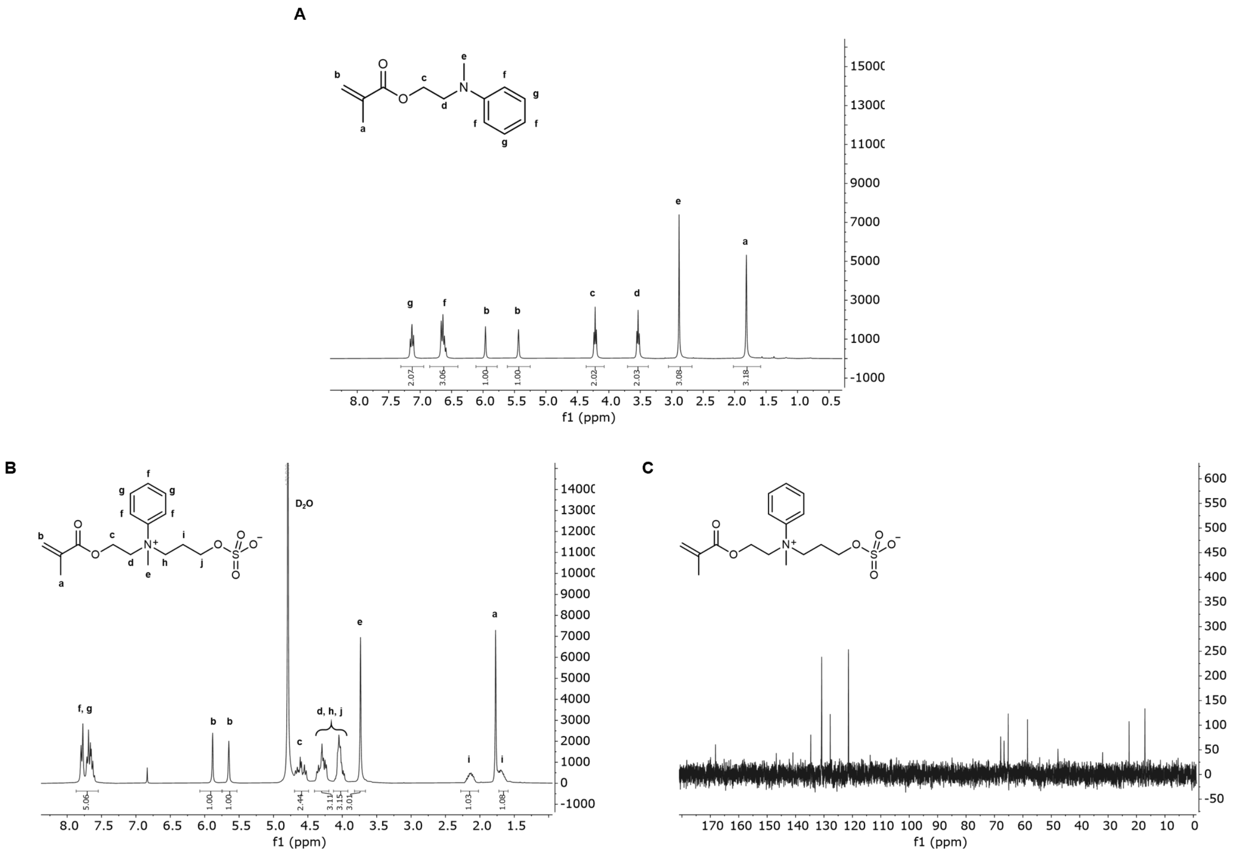

Fig. S13: NMR spectra of 2-(methyl(phenyl)amino)ethyl methacrylate (A) and ZB5 (B, 1H NMR and C, 13C NMR).

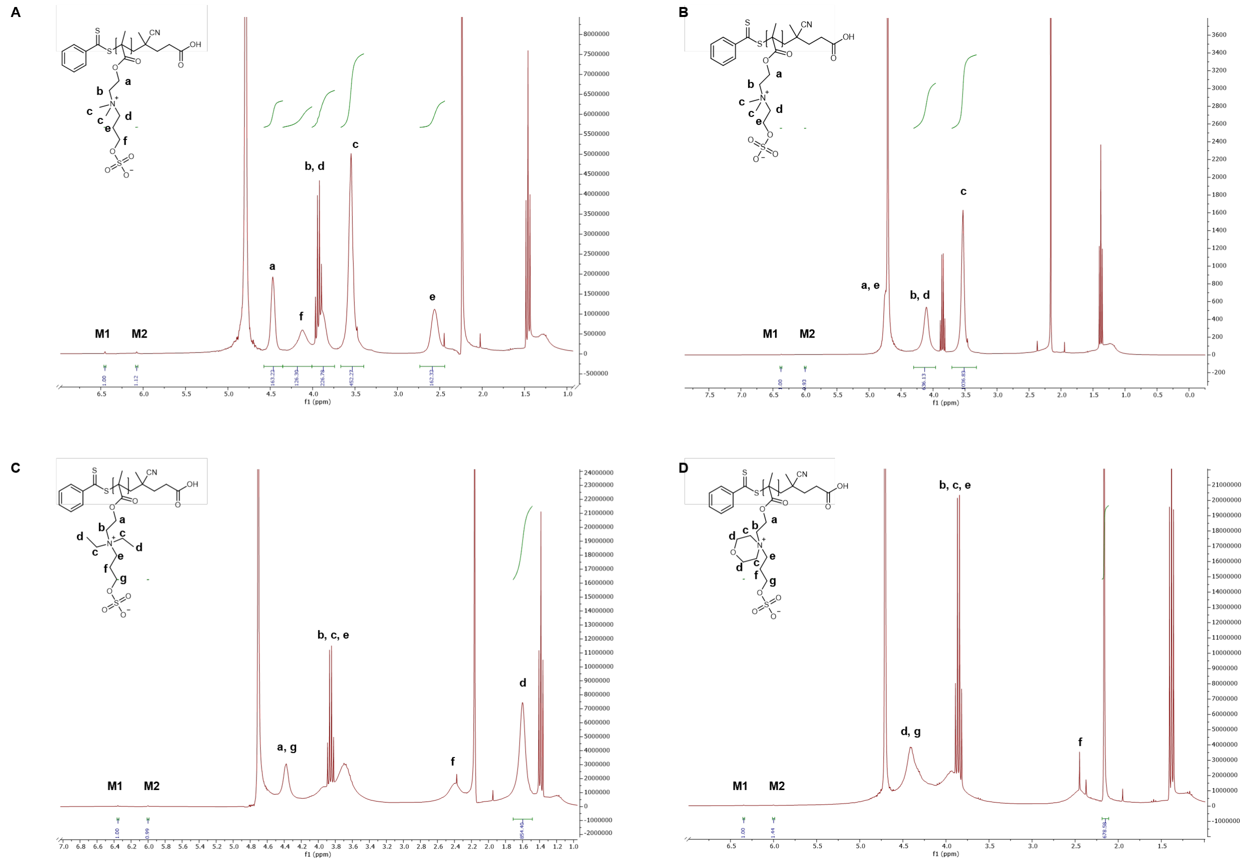

Fig. S14: NMR spectra of homopolymers of ZB, ZB2, ZB3, and ZB4 after polymerization.

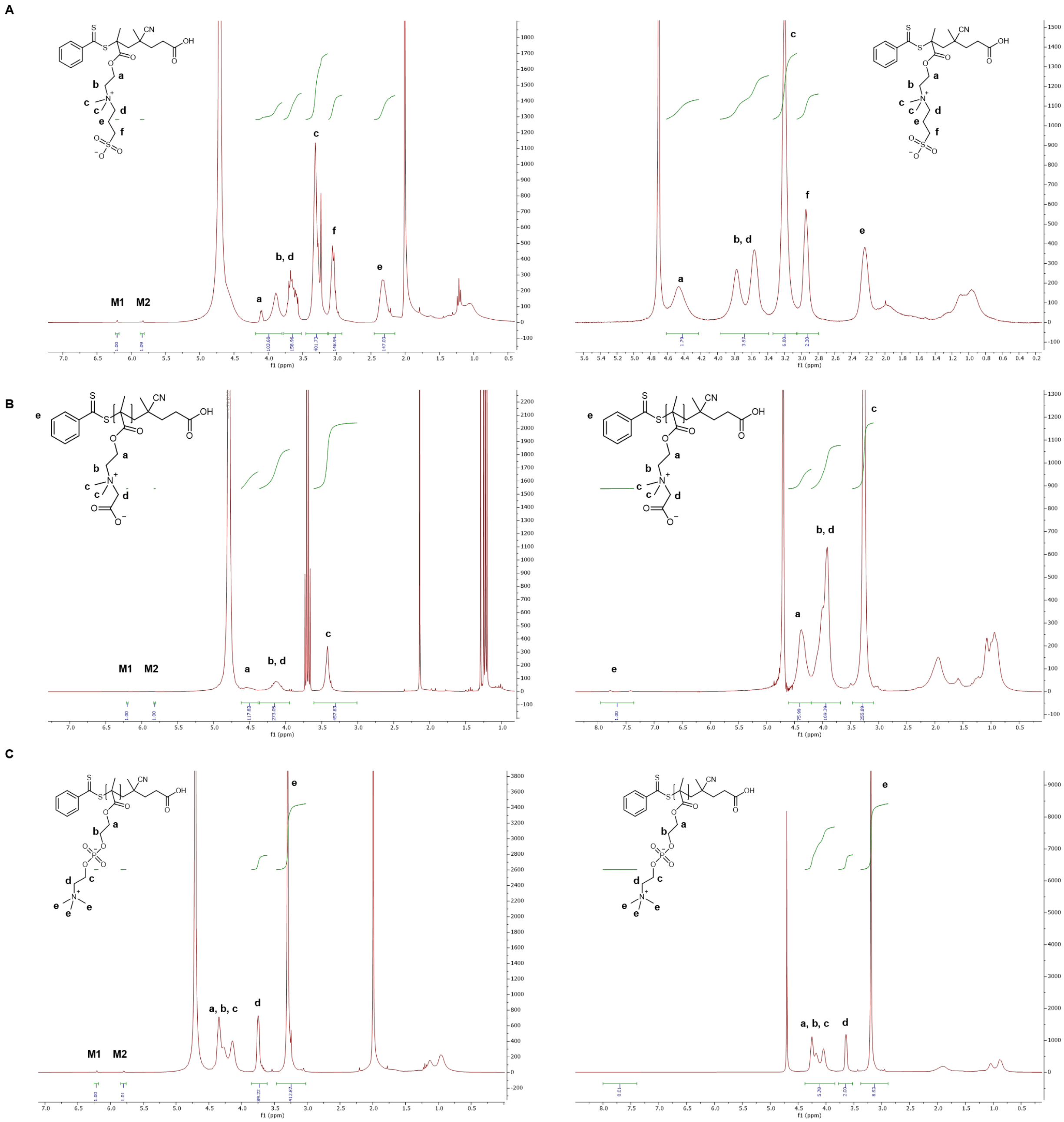

Fig. S15: NMR spectra of homopolymers of CB, SB, and MPC after polymerization (left) and after purification (right).

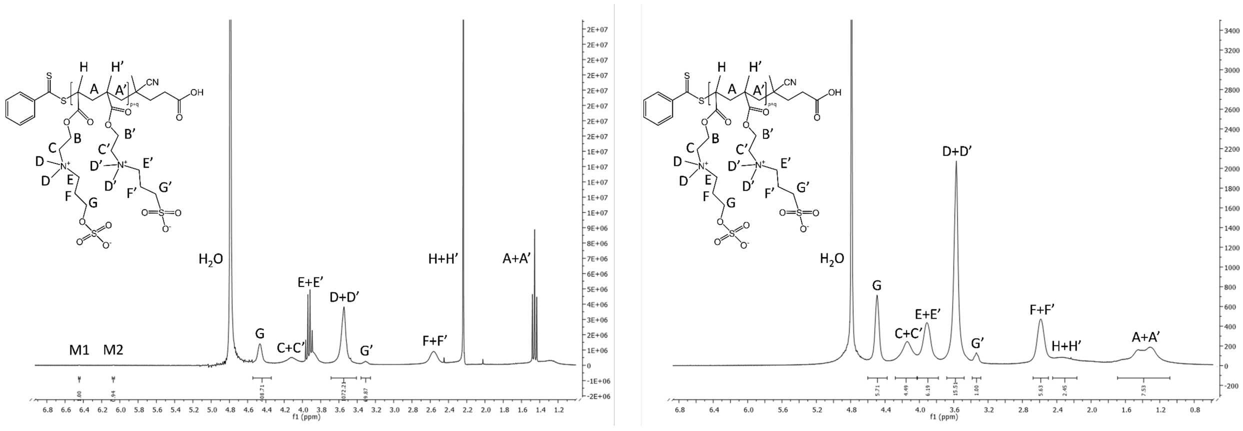

Fig. S16: NMR spectra of copolymer p(180ZB-co-20SB) after polymerization (left) and after purification (right).

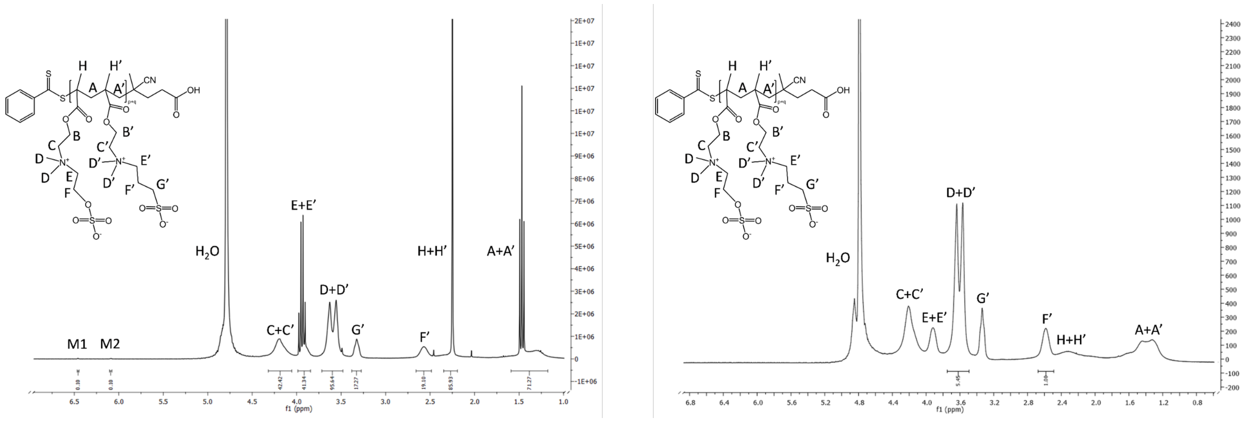

Fig. S17: NMR spectra of copolymer p(100ZB2-co-100SB) after polymerization (left) and after purification (right).

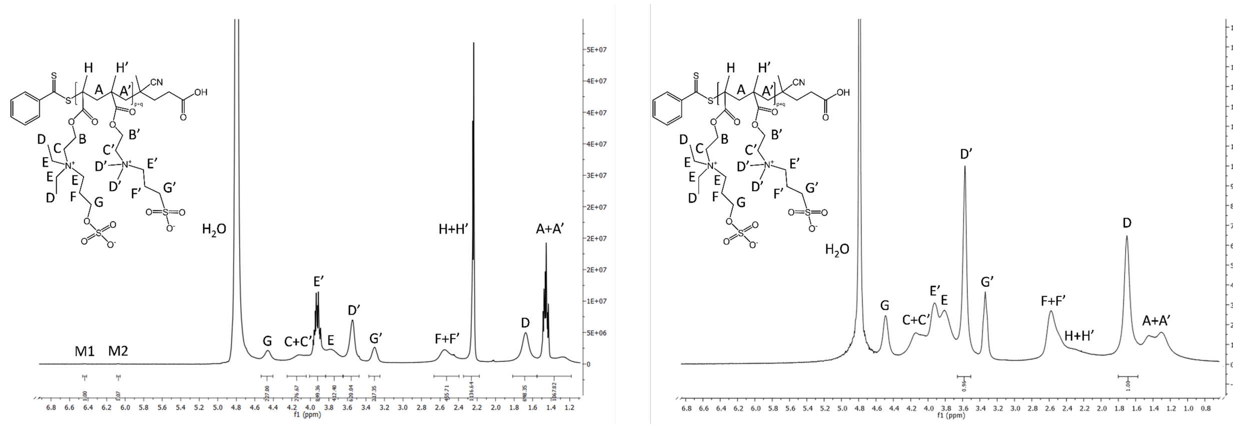

Fig. S18: NMR spectra of copolymer p(100ZB3-co-100SB) after polymerization (left) and after purification (right).

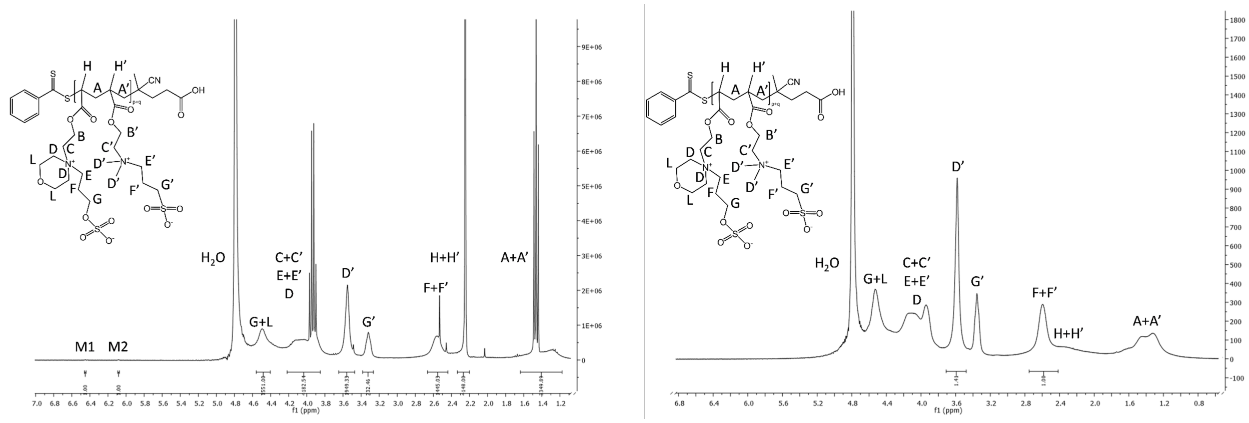

Fig. S19: NMR spectra of copolymer p(100ZB4-co-100SB) after polymerization (left) and after purification (right).

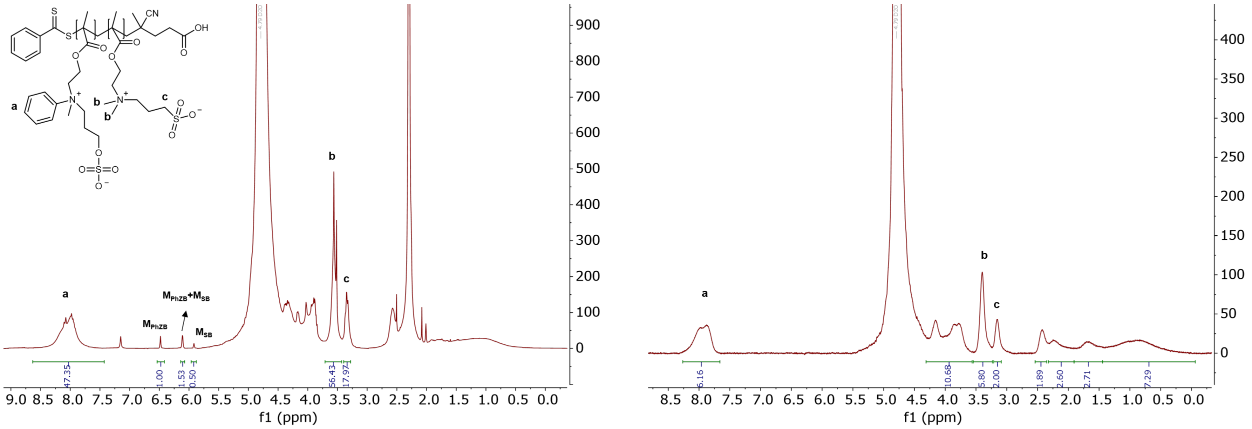

Fig. S20: NMR spectra of copolymer p(100ZB5-co-100SB) after polymerization (left) and after purification (right).

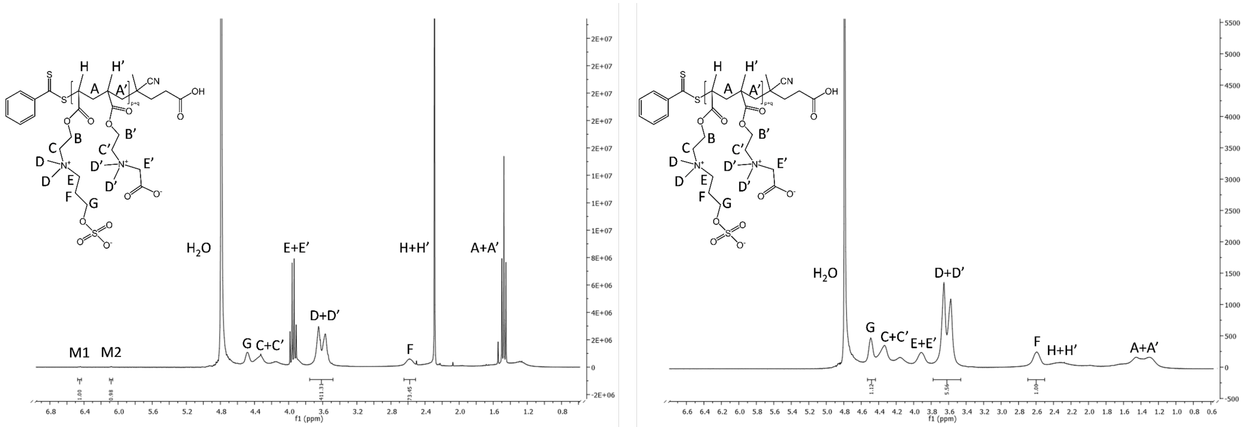

Fig. S21: NMR spectra of copolymer p(100ZB-co-100CB) after polymerization (left) and after purification (right).

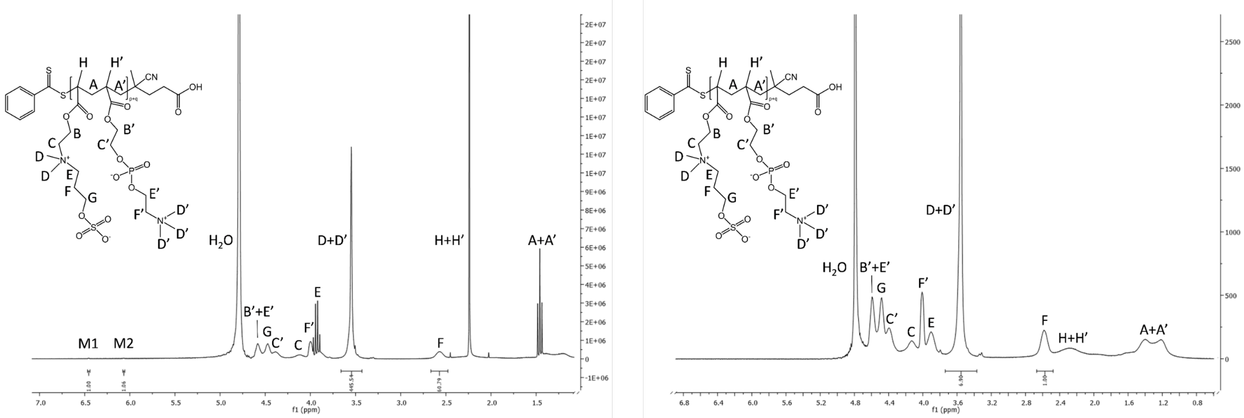

Fig. S22: NMR spectra of copolymer p(100ZB-co-100MPC) after polymerization (left) and after purification (right).

References

(1) Sponchioni, M.; Rodrigues Bassam, P.; Moscatelli, D.; Arosio, P.; Capasso Palmiero, U. Biodegradable zwitterionic nanoparticles with tunable UCST-type phase separation under physiological conditions. *Nanoscale* **2019**, *11*, 16582–16591.

(2) Gimondi, I.; Salvalaglio, M. CO2 packing polymorphism under confinement in cylindrical nanopores. *Mol. Syst. Des. Eng.* **2018**, *3*, 243–252.
